# A Density Functional Theory-Based Scheme to Compute the Redox Potential of a Transition Metal Complex: Applications to Heme Compound

**DOI:** 10.3390/molecules24040819

**Published:** 2019-02-25

**Authors:** Toru Matsui, Jong-Won Song

**Affiliations:** 1 Department of Chemistry, Graduate School of Pure and Applied Sciences, University of Tsukuba, 1-1-1 Tennodai, Tsukuba, Ibaraki 305-8577, Japan; 2 Department of Chemistry Education, Daegu University, Gyeongsan-si 113-8656, Korea

**Keywords:** metal complex, range-separated density functional theory (DFT), redox potential

## Abstract

We estimated the redox potential of a model heme compound by using the combination of our density functionals with a computational scheme, which corrects the solvation energy to the normal solvent model. Among many density functionals, the LC-BOP12 functional gave the smallest mean absolute error of 0.16 V in the test molecular sets. The application of these methods revealed that the redox potential of a model heme can be controlled within 200 mV by changing the protonation state and even within 20 mV by the flipping of the ligand histidine. In addition, the redox potential depends on the inverse of the dielectric constant, which controls the surroundings. The computational results also imply that a system with a low dielectric constant avoids the charged molecule by controlling either the redox potential or the protonation system.

Academic Editors: Yasutaka Kitagawa, Ryohei Kishi and Masayoshi Nakano

## 1. Introduction

Heme compounds play a key role in many biological systems, which bind an oxygen or control the electron transfer reactions. Heme, which has many kinds of compound (*a*, *b*, *c*, and *o*), commonly consists of iron ion, porphyrin, and two propionic acid groups. By controlling the surroundings, heme can control the redox potential. The redox potential of heme ranges from +400 mV to −400 mV and depends not only on the kind of hemeprotein but also on the solution pH [1]. The redox potential determines the property used for electronic devices and even controls the chemical reaction. The correlation between the structure and the function of a heme compound has been a hot research topic [2]. The amino acids which surround a heme compound can control its redox potential [3,4,5,6,7]. Positive/negative charge (basic/acidic residue) also affects the redox potential, and hydrophilic/hydrophobic residues also affect the dielectric constant around the heme compound. This environment necessitates an improved and systematic understanding of the surrounding effect.

In order to evaluate how the surroundings effectively control the redox potential, an approach from a quantitative computational scheme by quantum chemistry is also necessary. Since a heme contains an iron cation, a protein containing a heme is considered a kind of 3d-transition metal complex (TMC). Many protocols are available to compute the redox potential of a TMC [8,9,10,11,12]. However, there are three barriers in the way of estimating the redox potential in conventional density functional theory (DFT). First, the use of the standard hydrogen electrode (SHE) potential raises a problem. Many theoretical studies employ a different SHE potential (in many cases 4.24 V or 4.44 V), which influences the computed result. Although one study proposed an SHE potential by accurate computational scheme, the SHE potential used by conventional DFT cannot yet reproduce the SHE potential accurately [13,14]. Second, using polarizable continuum model (PCM) for an excessively charged system introduces severe errors. Third, employing DFT to the TMC can introduce some errors, because of an incomplete formation of the exchange/correlation functional.

To solve the first two problems, we proposed a “pseudo counter ion solvation” (PCIS) scheme as a calculation protocol [15]. In this scheme, the correction energy is added as the solvation energy of a pseudo counter ion. Moreover, the SHE potential is also considered a “parameter”. We revealed that such treatments could provide the experimental redox potential effectively. For the applications, this method enabled us to estimate the redox potential of a metal-containing protein, such as Cu-NiR [16] and Fe-S cluster [17]. To solve the third problem and to improve the computational accuracy, the range-separated DFT can be a powerful strategy to forecast the property of a molecule.

This paper reviews the PCIS scheme, with a special focus on the reason why such a correction is necessary, and presents its applicability to heme compounds in order to understand the surrounding effects. In Section 2, we briefly review our original theoretical/computational schemes. In Section 3, we discuss the computational results, and Section 4 presents the study’s conclusion.

## 2. Theory and Computational Scheme

### 2.1. PCIS Scheme

In our computational scheme, we directly estimated the redox potential in a solution, because we employed the result of geometry optimization in the self-consistent reaction field. Assuming a one-electron oxidation reaction (A(red) → A(ox) + e^−^), the redox potential of a TMC can be obtained by Equation (1), in accordance with Nernst’s law:

(1) $$E_{\mathrm{redox}}=\frac{G_{\mathrm{ox}}-G_{\mathrm{red}}}{F}-E_{\mathrm{SHE}}$$

where *F* is the Faraday constant, *G*_ox_ and *G*_red_ are the Gibbs free energies of both the oxidized and reduced states of the TMCs in a solution, respectively, and *E*_SHE_ is the SHE potential. 

In the supplementary materials, we present that *G*_ox_ − *G*_red_ can be approximated to the self consistent field (SCF) energy change in many compounds within the range of quantum chemical calculation. In this assumption, *G*_ox_ − *G*_red_ can be interpreted as the ionization potential with the geometry optimization (*E*_ox_ − *E*_red_), adiabatic ionization potential (AIP). This value is not exactly the AIP, because we assumed a redox reaction in the solvation. When the unit of energy is written in eV, the Faraday constant is considered as 1, and Equation (1) is modified as follows:

*E*_redox_ = (*E*_ox_ − *E*_red_) − *E*_SHE_ = *E*_AIP_ − *E*_SHE_ (2)

However, a continuum model without any correction underestimates (or overestimates) the redox potentials for excess positive (or negative) systems. We added a correction term to the energies for both the oxidized and reduced states in the atomic unit, as shown in Equations (3) and (4):

(3) $$E_{\mathrm{ox}}^{\mathrm{corr}}=E_{\mathrm{ox}}-\frac{Eq^{2}}{2R_{\mathrm{ox}}}\left(1-\frac{1}{\epsilon_{r}}\right)\mathrm{erf}\left(\mu r_{\mathrm{ox}}\left|q\right|\right)$$

(4) $$E_{\mathrm{red}}^{\mathrm{corr}}=E_{\mathrm{red}}-\frac{E{\left(q-1\right)}^{2}}{2R_{\mathrm{red}}}\left(1-\frac{1}{\epsilon_{r}}\right)\mathrm{erf}\left(\mu r_{\mathrm{red}}\left|q-1\right|\right)$$

where *ε*_r_ and *q* are the dielectric constant and the charge of the oxidized state, respectively. We introduced a multiplying term *E* (=27.211 eV/a.u.) for clarity, because the unit of correction terms should be aligned to eV. *V* is the volume of the cavity for a given TMC at the optimized geometry from the PCM calculation. Assuming that the cavity is treated as a sphere with an approximate radius of *r*, then *R*_ox_ or *R*_red_ is proportional to the approximate radius of the cavity sphere:

(5) $$R=ar=a\sqrt[{3}]{\frac{3V}{4\pi }}\;(a=\mathrm{const}.)$$

The effect of the charge correction decreases as the system becomes larger. In many cases, the geometries are relatively unaffected throughout the redox reaction, i.e., *r*_red_ ≈ *r*_ox_ = *r*. When we replace *E*_ox_/*E*_red_ with the corrected energy, the corrected term can be written as follows:

(6) $$E_{\mathrm{redox}}=E_{\mathrm{AIP}}-\frac{E}{2ar}\left(1-\frac{1}{\epsilon_{r}}\right)\{q^{2}\mathrm{erf}(\mu r\left|q\right|)-{\left(q-1\right)}^{2}\mathrm{erf}(\mu r\left|q-1\right|)\}-E_{\mathrm{SHE}}$$

The parameters *a*, *μ*, and *E*_SHE_ should be determined by several methods. In accordance with our previous study, we employed 39 TMCs as the test molecules with the experimental redox potential. (See the supplementary materials in the reference and the references within.) Actually, we prepared *E*_redox_ (*E*_exp_) and the charge for both of the reduced/oxidized states. From the computed results, *E*_AIP_ and *r* are obtained by Equations (2) and (5). 

### 2.2. LC-BOP12, LC-BOP12, LCgau-BOP, and LCgau-BOP12

In the long-range correction (LC) scheme [18,19,20,21,22], the electron repulsion operator, 1/*r*_12_, is divided into short- and long-range parts using a standard error function,

(7) $$\frac{1}{r_{12}}=\frac{\mathrm{erf}(\mu r_{12})}{r_{12}}+\frac{1-\mathrm{erf}(\mu r_{12})}{r_{12}}$$

where *r*_12_ = | **r**_1_ – **r**_2_ | for the coordinate vectors of electrons **r**_1_ and **r**_2_, and *μ* is the parameter which determines the proportion between the two ranges depending on the value of *r*_12_. The first term of Equation (7) is the long-range interaction term, through which the long-range orbital–orbital exchange interaction is described using the Hartree-Fock (HF) exchange integral, such as

(8) $$E_{x}^{lr}=-\frac{1}{2}\sum_{\sigma }\sum_{i}^{occ}\sum_{j}^{occ}\int \int \psi_{i\sigma }^{*}\left(r_{1}\right)\psi_{j\sigma }^{*}\left(r_{1}\right)\frac{\mathrm{erf}\left(\mu r_{12}\right)}{r_{12}}\psi_{i\sigma }\left(r_{2}\right)\psi_{j\sigma }\left(r_{2}\right)d^{3}r_{1}d^{3}r_{2}$$

where $\psi_{i\sigma }^{}$ is the *i*th σ-spin molecular orbital. The DFT [23] exchange functional is included through the second term of Equation (7), which is multiplied by the square of the one-particle density matrix for a uniform electron gas and is then integrated. Therefore, the short-range part of the exchange integral can be incorporated by modifying the usual exchange integral form,

(9) $$E_{x}=-(1/2)\Sigma_{\sigma }\int \rho_{\sigma }^{4/3}K_{\sigma }d^{3}R$$

into

(10) $$E_{x}^{sr}=-\frac{1}{2}\sum_{\sigma }\int \rho_{\sigma }^{4/3}K_{\sigma }\times \left\{1-\frac{3}{8}a_{\sigma }\left[\sqrt{\pi }\mathrm{erf}\left(\frac{1}{2a_{\sigma }}\right)+2a_{\sigma }(b_{\sigma }-c_{\sigma })\right]\right\}d^{3}R$$

where $a_{\sigma }$, $b_{\sigma }$, and $c_{\sigma }$ are defined as

(11) $$a_{\sigma }=\frac{\mu }{6\sqrt{\pi }}\rho_{\sigma }^{-1/3}K_{\sigma }^{1/2}$$

(12) $$b_{\sigma }=\exp\left(-\frac{1}{4a_{\sigma }^{2}}\right)-1$$

and

(13) $$c_{\sigma }=2a_{\sigma }^{2}b_{\sigma }+\frac{1}{2}$$

The use of *K_σ_* allows the incorporation of generalized gradient approximation (GGA) functionals. The DFT functionals combined with the LC scheme have been successfully applied to larger systems and properties which demand correct descriptions of inter-electronic long-range distances [24,25,26,27,28,29,30,31,32,33]. 

The LC scheme with the short-range Gaussian attenuation showed greatly improved features for the LC scheme, and we termed this the LCgau scheme [34], where the electron repulsion operator, 1/*r*_12_, was divided using a standard error function augmented by an additional Gaussian function,

(14) $$\frac{1}{r_{12}}=\frac{\mathrm{erf}(\mu r_{12})}{r_{12}}+k\frac{2\mu }{\sqrt{\pi }}e^{-(1/a)\mu^{2}r_{12}^{2}}+\frac{1-\mathrm{erf}(\mu r_{12})}{r_{12}}-k\frac{2\mu }{\sqrt{\pi }}e^{-(1/a)\mu^{2}r_{12}^{2}}$$

The DFT exchange functional is included through the first two terms of Equation (14), and the HF exchange integral is included through the second two terms. The *μ*, *a*, and *k* parameters were previously determined to be 0.42, 0.011, and 18.0, respectively, to give the lowest sum of the root mean square (rms) errors of the atomization energies and the barrier heights.

The one-parameter progressive (OP) correlation functional [35] is an approximated functional based on the Colle–Salvetti (CS) correlation functional [36] as the Lee-Yang-Par (LYP) [37] correlation functional. The OP correlation functional has the form

(15) $$E_{c}^{\mathrm{OP}}=-\int \rho_{\alpha }\rho_{\beta }\frac{1.5214\beta_{\alpha \beta }+0.5764}{\beta_{\alpha \beta }^{4}+1.1284\beta_{\alpha \beta }^{3}+0.3183\beta_{\alpha \beta }^{2}}d^{3}R$$

where

(16) $$\beta_{\alpha \beta }=q_{\mathrm{OP}}^{\alpha \beta }\frac{\rho_{\alpha }^{1/3}\rho_{\beta }^{1/3}K_{\alpha }K_{\beta }}{\rho_{\alpha }^{1/3}K_{\alpha }+\rho_{\beta }^{1/3}K_{\beta }}$$

Here, $q_{\mathrm{OP}}^{\alpha \beta }$ is a semi-empirical parameter that determines the correlation length, and the function *K_σ_* is defined as Equation (9). As shown in Equations (15) and (16), two factors, $q_{\mathrm{OP}}^{\alpha \beta }$ and *K_σ_*, are specifically included in the OP correlation functional, which results in a strong dependency on the combined exchange functional formulation. In the case of the LC-Becke88 exchange (B88) [38] and the OP correlation functional (LC-BOP), $q_{\mathrm{OP}}^{\alpha \beta }$ = 2.367 was used, which was determined to reproduce the exact correlation energy of the carbon atom with *K_σ_* of the B88 exchange,

(17) $$K_{\sigma }=3{\left(\frac{3}{4\pi }\right)}^{1/3}+2\zeta \frac{x_{\sigma }^{2}}{1+6\zeta x_{\sigma }\sinh^{-1}x_{\sigma }},$$

where *x_σ_* = $\left|\nabla \rho_{\sigma }\right|/\rho_{\sigma }^{4/3}$ and ς = 0.0042 [39]. However, we found that a slight change of $q_{\mathrm{OP}}^{\alpha \beta }$ to 2.46 improved the performance of LC-BOP with *μ* = 0.42 [39] on the thermochemical properties as well as the reaction barrier heights, which we named LC-BOP12 [39]. Moreover, the so-called LCgau-BOP12 [40], which had the new parameter set of *μ* = 0.42, *a* = 0.04, *k* = 2.0, and $q_{\mathrm{OP}}^{\alpha \beta }$ = 2.42, showed an improved and balanced performance for thermochemical properties.

### 2.3. Computational Details

Throughout this study, the standard version of the GAUSSIAN09 program package [40] was used for the calculation. While using the LC and LCgau exchange functional and/or the one-parameter progressive (OP), LC-BOP, LC-BOP12, LCgau-BOP, LCgau-BOP12, and LCgau-B97 [41] functionals developed by one of the authors, the computation was performed by a modified version of GAUSSIAN09. We employed a conductor-like polarizable continuum model (C-PCM) [42,43,44] with the correction as a solvation model and universal force field (UFF), which is the default of GAUSSIAN09, as a model for the cavity space. All geometries were optimized for each density functional or basis set. The reference state was in the condition of 25 °C (298.15 K) and 1 atm. For the basis set, Stuttgart/Dresden double zeta (SDD) with effective core potential for metal atoms and 6-31++G(d,p) for the other atoms (H, C, N, O, S, and Cl) was used in this study. For exceptional calculations, Truhlar’s continuum solvent model (SMD) [45] was also used (see Section 3.1.2). The parameters used in the PCIS scheme were fitted by Mathematica. As shown in the Supplementary Materials, the estimation can be carried out by a spreadsheet program. 

In some computational methods, we could not obtain the optimized geometries of some TMCs, because the SCF did not converge under any conditions. We removed such complexes for the fitting. In the Supplementary Materials, we list the complexes that were not optimized. 

## 3. Results and Discussion

### 3.1. Significance of the Correction Term

In this subsection, we discuss the necessity of the correction term discussed above. Here, we divide the problem into the HF exchange parameter and the solvation model. For the former, many computational papers, which compute the redox potential of TMCs, have employed “the best” functional and “the best” SHE potential that could reproduce the experimental redox potential. Actually, the value *E*_AIP_ strongly depends on the computational method. For the solvation model, such as the PCM, many papers have shown that it introduces some errors. Solvent model dependency should be investigated, including the case without the solvent model. Here, we evaluated the accuracy of the redox potential by using Equation (2).

#### 3.1.1. HF Exchange vs. Redox Potential

**Figure 1:**
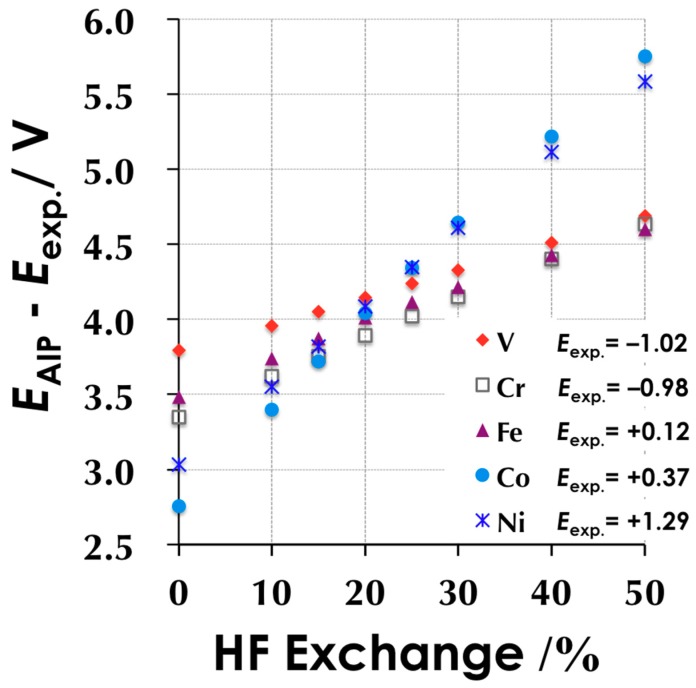
The dependency of *E*_AIP_ − *E*_exp_ on the HF ratio for the M^II/IIII^ (EDTA) complexes. (M = V, Cr, Fe, Co, and Ni.) The experimental redox potential (in V) is listed in the figure.

To investigate the HF exchange dependency on the ionization potential of a TMC, we plotted the HF exchange to the *E*_AIP_ − *E*_exp_ of 3d-metal ethylenediaminetetraacetic acid (EDTA) complexes in Figure 1. Judging from Equation (2), the value *E*_AIP_ − *E*_exp_ should be the same as *E*_SHE_, when we employ a similar compound with the same net charge. *E*_SHE_ can be estimated as an average of the value *E*_AIP_ − *E*_exp_ of five species. For simplicity, we controlled only the HF ratio of B3LYP, i.e., the parameter A in the following equation was changed in this plot:

*E*_Mod.-B3LYP_ = A *E*_X_^Slater^ + (1 − A) *E*_X_^HF^ + B Δ*E*_X_^Becke^ + C *E*_C_^LYP^ + (1 − C) *E*_C_^VWN^ (18)

According to Figure 1, the linear dependency on the HF exchange ratio can be found in TMCs. The pure functional (HF = 0%, close to the BLYP functional) significantly underestimates the *E*_AIP_ values of Co(EDTA) and Ni(EDTA), whereas the high ratio (HF = 50%, close to the BHandHLYP functional) overestimates them. The difference is explained by the tendencies of DFT. A pure GGA functional underestimates the Highest occupied molecular orbital-Lowest unoccupied molecular orbital (HOMO–LUMO) gap and the ionization potential of a molecule, whereas HF overestimates them. Many researchers naturally choose the value 4.2–4.3 V for *E*_SHE_ and the functional whose HF ratio is around 25%, because some papers reported a value of 4.24 V for the *E*_SHE_. One of the authors previously computed a value for the *E*_SHE_ of 4.48 V [14], whereas Figure 1 gives a corresponding HF ratio of 30–40%. Such a functional introduces a large error. Although normal B3LYP with 4.03 V for *E*_SHE_ introduces the smallest error, the choice of *E*_SHE_ is not practical, because few papers have reported the *E*_SHE_ around 4.0 V. Within the adiabatic HF exchange mixture, 15–25% HF exchange optimizes the performance in reproducing the redox potential of TMCs. Interestingly, the gradient of *E*_AIP_ − *E*_exp_ against the HF ratio strongly depends on the kind of 3d-metal ion. The gradient is in the order of V < Cr < Fe < Co < Ni, which correlates to the number of 3d-electrons.

Note here that the value *E*_AIP_ − *E*_exp_ is underestimated in these calculations because the net charge of M(EDTA) is −1/−2 for the oxidized/reduced state. Judging from Equations (3) and (4), the correction term should be positive. Actually, the fitted *E*_SHE_ for B3LYP is 4.22 V (see next page), a little larger than the value mentioned above. 

#### 3.1.2. Solvation Model Dependencies

The PCIS scheme is based on the energy correction to the continuum model which does not describe the solvation energy in a charged system. This section presents the solvent model dependencies, including the case without the solvent model (gas phase). Since many papers have reported that the solvent model (SMD) describes the solvent energy effectively, SMD was also added to the test calculation. 

Table 1 lists the average and deviation values determined for the value *E*_AIP_ − *E*_exp_ for each net charge ranging from −3 to +3. A calculation without any solvent model does not make sense to estimate the redox potential, especially in the excess charged system, for two reasons. First, regarding the average of *E*_AIP_ − *E*_exp_, there was a 20 V difference between the system with a “−3” charge and that with a “+3” charge. Second, the deviation was larger than that in the case of using a solvent model. 

The difference between the SMD and the C-PCM was the variation of the charge dependencies on *E*_AIP_ − *E*_exp_. The average value of the SMD ranged from 3.95 V to 4.54 V, whereas the C-PCM ranged from 3.80 to 5.12 V. Since an error from the excess charge is inevitable in any solvent model, it is necessary to correct them. Our PCIS scheme can solve these problems by using two parameters, *a* and *μ*.

### 3.2. Functional Dependencies on Redox Potential

In this section, we employed several DFTs to assess the performance for computing the redox potential in 38 complexes (same as the test molecules for the fitting). DFT can be divided into: (A) the hybrid DFT (B3LYP, BLYP, PBE, BHandHLYP B3PW91, and M06) and (B) the range-separated DFT (ωB97XD, LC-BLYP, LC-BOP, LC-BOP12, LCgau-BOP, LCgau-BOP12, LC-ωPBE, and LCgau-B97). 

Table 2 and Table 3 list the optimized parameters and mean absolute error (MAE) in the test set. B3LYP gave the smallest MAE among the hybrid DFTs, which means that the LYP correlation functional was slightly better than the PW91 within this test set. The fitted *E*_SHE_ also increased when the HF ratio increased. The error of using BHandHLYP is mainly caused in cobalt/nickel complexes (max. 1.46 V in Co(EDTA)). The pure functional underestimated the *E*_SHE_, while the MAE was not large compared with BHandHLYP or M06. In the case of the range-separated DFT, the difference was unremarkable. Neither the range-separation parameter nor the correlation function affected either the SHE potential or MAE. Among these calculations, LC-BOP12 presented the best performance, with a MAE of 0.16 V in the test of 38 complexes. Some papers focus on specific metal species for computing the redox potential. Actually, the maximum error in many functionals is from a cobalt or nickel compound. For example, Hughes and Friesner added the parameter for a nickel complex [46]. Knapp’s group focused on Fe/Mn/Ni complexes to reproduce the redox potential [47]. To reduce the MAE, such a treatment could be necessary for the next stage.

### 3.3. Application to Heme Products

By using the LC-BOP12 functional, we next tackled the redox potential of heme products. Takano’s group investigated patterns for the coordination of the iron using the nonredundant structural data [48,49]. They reported that the axial ligand “histidine (His)/His” accounts for 23% in 10,748 independent structures. In this section, the 5th and 6th ligands were fixed to the 5-methyl imidazole under the assumption of the model His. Throughout this section, we carried out the geometry optimization by LC-BOP12/6-31++G(d,p) with SDD, which gave the smallest MAE in the test molecular set. We assumed the doublet (S = +1/2) for the oxidized state and the singlet (S = 0) for the reduced state, in accordance with previous computational studies [5,6,48]. 

#### 3.3.1. Flipping of the Model Histidine

**Figure 2:**
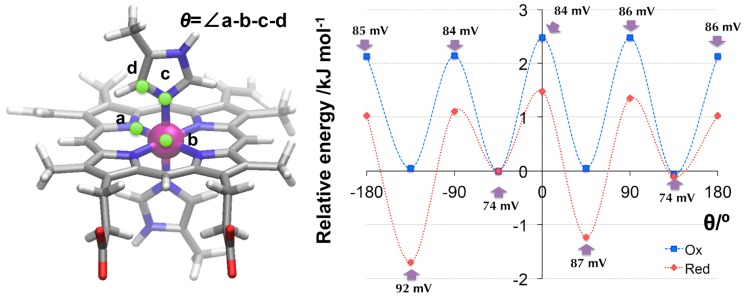
The potential energy surface of the oxidized/reduced state in heme *b*. The zero-point is set at θ = −45°. The local maxima at the potential energy surface are the transition states for the histidine flipping.

Firstly, the effect of flipping in 5-methyl imidazole to the redox potential was investigated for the model His. The imidazole ring was rotated so that the value *θ* changed. Figure 2 shows the potential energy surface for the dihedral. 

The redox potential also changed when the imidazole ring flipped. The value ranged from +74 mV to +92 mV vs. SHE potential. When *θ* was −135° or 45°, the reduced state was more stabilized than the oxidized state. Even the flipping of histidine changed the redox potential by about 20 mV. Moreover, the energy difference was very low at less than 3 kJ/mol. The thermal energy allowed the histidine to flip easily and controlled the redox potential.

#### 3.3.2. Kind of Heme vs. Redox Potential

**Figure 3:**
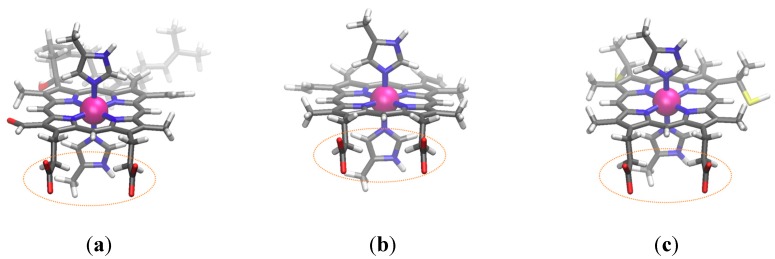
The optimized geometries of heme compounds: (**a**) heme *a*, (**b**) heme *b*, and (**c**) heme *c* (the cysteine part is replaced with thiol group). The circled area is propionic acid (-CH_2_-CH_2_-COOH), which can control the protonation state.

There are many varieties of heme compounds (such as heme *a*, *b*, *c*, and *o*), and the kind of heme affects the redox potential. We computed the redox potential of three patterns of heme compounds—heme *a*, *b*, and *c*. (See Figure 3 for the structure of each compound). Since a heme has two propionic acid parts, it can control the charge state by using protonation/deprotonation. Table 4 lists the computed redox potential.

Although the protonation increased the *E*_AIP_ in all compounds, it did not always increase the redox potential. Since the p*K*_a_ of a normal propionic acid is 4.95 in water, the heme can own a proton in the propionic acid part at low pH. Although our calculation cannot be compared directly with the experimental value, the results are within the range of the observable values.

#### 3.3.3. Dielectric Constants vs. Redox Potential

We also investigated the dielectric constant dependency on the redox potential of the electron transport enzyme, such as heme *c*. This application could act as an index of how the redox potential of heme compounds is affected by their surroundings. The dielectric constant *ε*_r_ in the case of being surrounded by proteins can range from 4 to 20, which corresponds to the region 1/*ε*_r_ = 0.05–0.25. 

**Figure 4:**
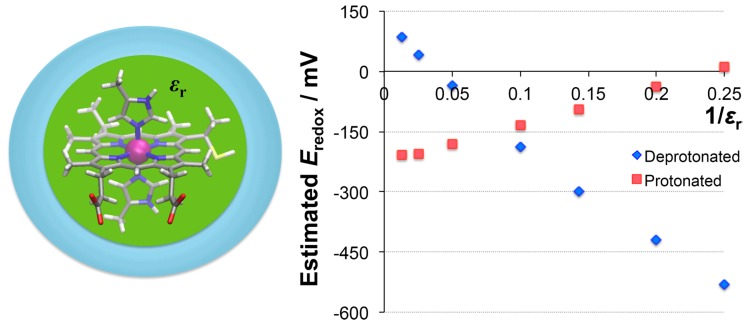
A plot of the redox potential against the inverse of *ε*_r_ in heme *c*. In both states, the correlation is nearly linear.

Figure 4 reveals a linear correlation between the redox potential and the inverse of the dielectric constant. In the case of the deprotonated state, the heme compounds tended to take an electron in a hydrophilic environment (high *ε*_r_), because the computed potential was positive. In a hydrophobic environment (low *ε*_r_), heme compounds released an electron due to the negative potential. 

In the protonated state, in contrast, the redox potential increased in a hydrophobic environment. The gradient of increase in the protonated state was smaller than the gradient of decrease in the deprotonated state. The changes of net charge through the oxidation reaction were −2/−1 for the deprotonated state and 0/+1 for the protonated state. A hydrophobic environment tends to avoid the charged system because of a Coulombic interaction. In this circumstance, the heme tended to be oxidized in the deprotonated state (which decreased the redox potential) but reduced in the protonated state (which increased the redox potential). Therefore, the net charge is also an important factor for controlling the redox potential.

## 4. Conclusions

We have presented the significance of correction to the solvation energy in the solvent model. In using a hybrid functional, the ratio of HF exchange plays an important role in determining the *E*_SHE_. With regard to a Co or Ni complex, a functional with a high HF exchange rate cannot describe the ionization potential as it can for other metal species and introduces critically large errors. In assessing the functionals, the LC-BOP12 functional provided the smallest MAE (0.16 V) in the test molecules. Although the improvement was small, it shows that any range-separated DFT can describe the redox potential effectively, regardless of the range-separation parameter. 

The application of the LC-BOP12 functional revealed that even the flipping of histidine can change the redox potential by about 20 mV. The protonation of the propionic acid of a heme compound can control the net charge, which in turn can control the redox potential within 300 mV. Among the model heme compounds, all types reduced the redox potential through protonation. Since the dielectric constant determines whether the system is hydrophilic or hydrophobic, this circumstance can be simulated by a PCM calculation by changing the dielectric constant. The results revealed a linear correlation between the redox potential and the inverse of the dielectric constant. The hydrophobic environment prefers not to be a charged system and controls the net charge. 

Further investigation of the surrounding effects will require the consideration of a larger molecular model, including the residues around a heme compound. As the model used in this study was limited to a heme compound and two histidines, we will tackle the effects of the surrounding molecules on the model in our future studies. 

## Figures and Tables

**Table 1:** The charge dependencies on the average and the deviation for the value *E*_AIP_ − *E*_exp_ (in eV).

Charge at Ox	N ^1^	Gas		SMD		C-PCM	
		Ave.	Dev.	Ave.	Dev.	Ave.	Dev.
−3	6	−7.12	1.30	3.95	0.43	3.55	0.34
−2	2	−3.84	0.84	3.96	0.09	3.80	0.18
−1	15	−0.49	0.67	4.23	0.55	4.24	0.27
0	4	2.69	0.44	4.26	0.21	4.31	0.29
1	5	5.73	0.70	3.94	0.22	4.23	0.29
2	2	8.90	0.25	4.18	0.33	4.61	0.30
3	4	13.57	0.35	4.54	0.44	5.12	0.39

^1^ Number of samples.

**Table 2:** The parameters of the pseudo counter ion solvation (PCIS) scheme and mean absolute error (MAE) by hybrid DFTs.

	*a*	*μ*	*HF Mix/%*	*E*_SHE_	MAE
BLYP ^1^	14.99	0.1364	0	3.76	0.22
PBE	18.67	0.1213	0	3.80	0.22
B3LYP ^1^	15.26	0.0383	20	4.26	0.17
BHandHLYP	6.21	0.0178	50	4.62	0.61
B3PW91	12.58	0.0361	20	4.19	0.18
M06 ^1^	11.55	0.0293	27	4.20	0.33

^1^ Taken from [15].

**Table 3:** The parameters of the PCIS scheme and MAE by range-separated DFTs.

	*a*	*μ*	RS Param. ^1^	*E*_SHE_	MAE
ωB97XD	7.57	0.0153	0.20	4.33	0.22
LC-BLYP ^2^	10.76	0.0226	0.47	4.46	0.20
CAM-B3LYP	11.83	0.0245	0.33	4.38	0.20
LC-BOP	11.53	0.0354	0.47	4.41	0.18
LC-BOP12	12.13	0.0364	0.42	4.25	0.16
LCgau-BOP	11.55	0.0293	0.42	4.20	0.18
LCgau-BOP12	8.97	0.0223	0.42	4.29	0.20
LC-ωPBE	12.07	0.0355	0.40	4.44	0.22
LCgau-B97	12.13	0.0365	0.20	4.44	0.21

^1^ Range-separation parameter expressed by *µ* or *ω*. ^2^ Taken from [15].

**Table 4:** The adiabatic ionization potential and estimated redox potential for each compound.

	Deprotonated		Protonated	
	*E*_AIP_^1^	*E*_redox_^2^	*E*_AIP_^1^	*E*_redox_^2^
Heme *a*	4.10	+0.17	4.21	−0.12
Heme *b*	3.98	+0.07	4.10	−0.23
Heme *c*	4.01	+0.09	4.12	−0.21

^1^ In eV. ^2^ In V.

## Supplementary Materials

The following are available online, Figure S1: The scatter plot for computed *E*_AIP_ and Δ*G*, Figure S2: Screenshot of “Microsoft Excel” (example for B3LYP), Table S1: Gibbs energy and SCF energy at the optimized structure and corresponding values, Table S2: The rate of HF exchange and its adiabatic ionization potential (in eV)’ Table S3: Electronic state for each compound assumed in this study, and the experimental data’ Tables S4–14: Detailed data for DFT comparison. This supplementary Materials include optimized geometries for the heme compounds, a sample input for Gaussian.
