# Supplementary material for: A Density Functional Theory-Based Scheme to Compute the Redox Potential of a Transition Metal Complex: Applications to Heme Compound
Source: Molecules. 2019 Feb 25;24(4):819. doi: 10.3390/molecules24040819 (PMC6412623; doi:10.3390/molecules24040819)
Supplement: Supplementary file 1 [file molecules-24-00819-s001.pdf]

Supplementary Materials for

A Density Functional Theory Based Scheme to Compute the Redox Potential of a Transition Metal Complex: Applications to Heme Compound

Toru Matsui [1,*] and Jong-Won Song [2,*]

[1] Department of Chemistry, Graduate School of Pure and Applied Sciences, University of Tsukuba, 1-1-1 Tennodai, Tsukuba, Ibaraki 305-8577, Japan; matsui@chem.tsukuba.ac.jp
[2] Department of Chemistry Education, Daegu University, Gyeongsan-si, 113-8656, Korea; sjoshua@daegu.ac.kr
* Correspondence: matsui@chem.tsukuba.ac.jp; Tel.: +81-29-853-7185; sjoshua@daegu.ac.kr; Tel.: +82-53-850-6989

Submitted to "Molecules"

Special Issue: Open-Shell Systems for Functional Materials

Contents:

Supporting Information 1: Gibbs energy vs. SCF energy

Supporting Information 2: The detailed data for M(EDTA)

Supporting Information 3: Electronic state for each compound assumed in this study, and the experimental data.

Supporting Information 4: How to obtain the redox potential (Caption of

Excel)

Supporting Information 5: Detailed data for DFT comparison

Supporting Information 6: The details of the data corresponding to section 3.3.1.

Supporting Information 7: The optimized geometries of heme compounds. (Corresponds to section 3.3.2)

Supporting Information 8: The details of the data corresponding to section 3.3.3.

Supporting Information 1: Gibbs energy vs. SCF energy

Table S1: Gibbs energy and SCF energy at the optimized structure and corresponding values.

	Oxidized		Reduced			
	Optimized E	Gibbs E	Optimized E	Gibbs E	E_{AIP}	ΔG
Co(cdta)	-1401.876	-1401.58852	-1402.047103	-1401.769721	4.656	4.931
Co(edta)	-1245.828265	-1245.630558	-1245.999799	-1245.813381	4.668	4.975
Co(h2o)2(phen)2	-1441.636829	-1441.283624	-1441.838791	-1441.500897	5.496	5.912
Co(ox)2(h2o)2	-1053.305021	-1053.240776	-1053.50468	-1053.457089	5.433	5.886
Co(ox)3	-1277.921741	-1277.886285	-1278.098012	-1278.076463	4.797	5.175
Co(pdta)	-1285.142731	-1284.916859	-1285.306854	-1285.094068	4.466	4.822
Cr(cdta)	-1343.105039	-1342.821542	-1343.21692	-1342.939612	3.044	3.213
Cr(cl)(l15)	-1295.637955	-1295.240766	-1295.755959	-1295.369054	3.211	3.491
Cr(CN)6	-644.6140782	-644.613762	-644.7070644	-644.708805	2.530	2.586
Cr(co)5(pycn)	-994.2842094	-994.204046	-994.3895167	-994.313571	2.866	2.980
Cr(cyclam)(cl)2	-1622.03706	-1621.714035	-1622.152638	-1621.839945	3.145	3.426
Cr(cyclam)(cn)2	-887.2478851	-886.913391	-887.3392611	-887.007088	2.486	2.550
Cr(edta)	-1187.057628	-1186.864377	-1187.167651	-1186.981884	2.994	3.197
Cr(en)2(cn)2	-653.8005152	-653.593593	-653.9031918	-653.70862	2.794	3.130
Cr(en)2(ncs)2	-1450.251947	-1450.047013	-1450.363981	-1450.171325	3.049	3.383
Cr(en)3	-658.3854453	-658.072907	-658.4945725	-658.19737	2.969	3.387
Cr(mida)2	-1188.264028	-1188.054752	-1188.370235	-1188.168485	2.890	3.095
Cr(tacn)2	-890.5852262	-890.164001	-890.6945886	-890.285175	2.976	3.297
Fe(bpy)(cn)4	-990.9447081	-990.799699	-991.1015355	-990.956054	4.267	4.255
Fe(bpy)2(cn)2	-1300.38134	-1300.093279	-1300.549764	-1300.263726	4.583	4.638
Fe(cdta)	-1379.981374	-1379.701815	-1380.131522	-1379.856009	4.086	4.196
Fe(cl)(l52)	-1673.035517	-1672.647945	-1673.214904	-1672.833131	4.881	5.039
Fe(CN)6	-681.5020455	-681.497077	-681.6494909	-681.643626	4.012	3.988
Fe(edta)	-1223.934842	-1223.744368	-1224.085726	-1223.901668	4.106	4.280
Fe(hbed)	-1459.322435	-1458.996435	-1459.456454	-1459.135382	3.647	3.781
Fe(nap)3	-1729.968361	-1729.634775	-1730.075647	-1729.746469	2.919	3.039
Fe(ox)3	-1256.042767	-1256.015906	-1256.187426	-1256.168876	3.936	4.162
Fe(py26diox)2	-1292.918491	-1292.729216	-1293.068155	-1292.877985	4.073	4.048
Fe(py2ox)	-1373.048445	-1372.782848	-1373.201409	-1372.937509	4.162	4.208
Mn(cn)5(no)	-698.821711	-698.822067	-698.9885344	-698.988804	4.539	4.537
Mn(CN)6	-661.9293441	-661.926886	-662.0522854	-662.050875	3.345	3.374
Ni(edta)	-1270.891361	-1270.697397	-1271.093711	-1270.903295	5.506	5.603
Ni(pdtc)	-2711.56069	-2711.432107	-2711.728416	-2711.60377	4.564	4.671
Ni(tacn)2	-974.4518234	-974.026888	-974.6448938	-974.22933	5.254	5.509
Ti(cdta)	-1314.333852	-1314.048389	-1314.48276	-1314.200316	4.052	4.134
Ti(edta)	-1158.288112	-1158.091703	-1158.436354	-1158.244806	4.034	4.166
Ti(hbed)	-1393.702168	-1393.371704	-1393.822766	-1393.495311	3.282	3.363
Ti(nap)3	-1664.375926	-1664.036952	-1664.475099	-1664.139777	2.699	2.798
V(edta)	-1171.764876	-1171.572274	-1171.876917	-1171.688415	3.049	3.160

This computation is performed at "B3LYP/6-31++G(d,p)" level. (SDD for metal elements. Solvent model: PCM-SMD)

The scatter plot is shown in Figure S1. Almost linear correlation is found at this computational level. When we use ΔG for the redox potential, the fitted SHE potential will be 0.1 V larger than the original PCIS scheme in accordance with the fitted equation. The intercept (shown as "0.0924" in Figure S1) is included in the E_{SHE} term in the approximation.

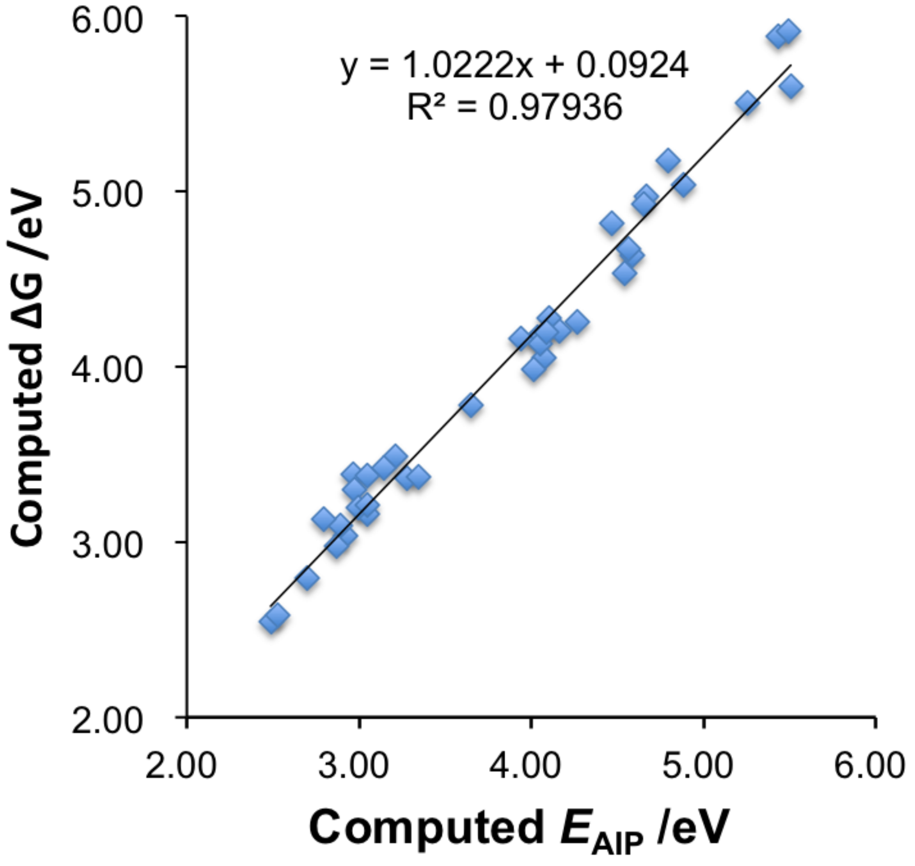

Figure S1: The scatter plot for computed E_{AIP} and ΔG.

Supporting Information 2: The detailed data for M(EDTA)

Table S2: The rate of HF exchange and its adiabatic ionization potential (in eV).

	0%			10%		
	Ox	Red	E_{AIP}	Ox	Red	E_{AIP}
V	-1169.203328	-1169.304508	2.75	-1170.47182	-1170.579084	2.92
Cr	-1184.495856	-1184.582965	2.37	-1185.766407	-1185.863384	2.64
Fe	-1221.34877	-1221.48106	3.60	-1222.627594	-1222.769344	3.86
Co	-1243.276602	-1243.391404	3.12	-1244.543143	-1244.681598	3.77
Ni	-1268.331049	-1268.489802	4.32	-1269.603152	-1269.781062	4.84

	15%			20%		
	Ox	Red	E_{AIP}	Ox	Red	E_{AIP}
V	-1171.109841	-1171.220437	3.01	-1171.750242	-1171.864248	3.10
Cr	-1186.405422	-1186.50737	2.77	-1187.046729	-1187.15371	2.91
Fe	-1223.271085	-1223.417865	3.99	-1223.916818	-1224.068584	4.13
Co	-1245.180687	-1245.330872	4.09	-1245.820834	-1245.982835	4.41
Ni	-1270.243343	-1270.431011	5.11	-1270.886109	-1271.083585	5.37

	25%			30%		
	Ox	Red	E_{AIP}	Ox	Red	E_{AIP}
V	-1172.392941	-1172.510384	3.20	-1173.037871	-1173.158736	3.29
Cr	-1187.690549	-1187.8023	3.04	-1188.336533	-1188.453057	3.17
Fe	-1224.565788	-1224.721454	4.24	-1225.216846	-1225.376184	4.34
Co	-1246.463927	-1246.63703	4.71	-1247.109497	-1247.293676	5.01
Ni	-1271.531741	-1271.7389	5.64	-1272.179856	-1272.396599	5.90

	40%			50%		
	Ox	Red	E_{AIP}	Ox	Red	E_{AIP}
V	-1174.334181	-1174.461749	3.47	-1175.638743	-1175.772712	3.65
Cr	-1189.635073	-1189.760774	3.42	-1190.942053	-1191.076335	3.65
Fe	-1226.526025	-1226.693157	4.55	-1227.844192	-1228.017597	4.72
Co	-1248.408267	-1248.613612	5.59	-1249.716889	-1249.941847	6.12
Ni	-1273.483727	-1273.718964	6.40	-1274.797348	-1275.049997	6.87

Note: See also Gaussian webpage: http://gaussian.com/dft/ (Accessed on January 14, 2019.) and "Keyword; Hybrid Functionals" tab, in which how to generate modified B3LYP functional is explained.

Supporting Information 3: Electronic state for each compound assumed in this study, and the experimental data.

Table S3: Electronic state for each compound assumed in this study, and the experimental data.

**Compound**	Charge_ox	Spin_ox	r_{ox}	Charge_red	Spin_red	Exp.
Co(cdta)	-1	1	8.77	-2	4	0.35
Co(edta)	-1	1	8.22	-2	4	0.37
Co(h2o)2(phen)2	3	1	9.46	2	4	0.68
Co(ox)2(h2o)2	-1	1	7.36	-2	4	0.78
Co(ox)3	-3	1	7.71	-4	4	0.57
Co(pdta)	-1	1	8.38	-2	4	0.36
Cr(cdta)	-1	4	8.81	-2	5	-0.96
Cr(cl)(l15)	2	4	8.76	1	5	-0.85
Cr(CN)6	-3	4	7.51	-4	3	-1.28
Cr(co)5(pycn)	0	1	8.16	-1	2	-1.03
Cr(cyclam)(cl)2	1	4	8.53	0	5	-0.75
Cr(cyclam)(cn)2	1	4	8.63	0	3	-1.12
Cr(edta)	-1	4	8.26	-2	5	-0.98
Cr(en)2(cn)2	1	4	7.87	0	5	-1.22
Cr(en)2(ncs)2	1	4	8.28	0	5	-0.90
Cr(en)3	3	4	8.01	2	5	-1.03
Cr(mida)2	-1	4	8.43	-2	5	-1.16
Cr(tacn)2	3	4	8.63	2	5	-1.14
Fe(bpy)(cn)4	-1	2	8.47	-2	1	0.54
Fe(bpy)2(cn)2	1	2	9.33	0	1	0.78
Fe(cdta)	-1	6	8.82	-2	5	0.04
Fe(cl)(l52)	2	6	9.47	1	5	0.81
Fe(CN)6	-3	3	7.44	-4	2	-0.24
Fe(edta)	-1	6	8.26	-2	5	0.12
Fe(hbed)	-1	6	9.30	-2	5	-0.37
Fe(nap)3	-3	6	10.02	-4	5	-0.58
Fe(ox)3	-3	6	7.78	-4	5	0.01
Fe(py26diox)2	-1	2	8.70	-2	1	0.20
Fe(py2ox)	0	2	9.14	-1	1	0.35
Mn(cn)5(no)	-2	4	7.39	-3	3	0.60
Mn(CN)6	-3	2	7.39	-4	1	0.36
Ni(edta)	-1	2	8.23	-2	3	1.29
Ni(pdtc)	-1	2	8.90	-2	3	0.38
Ni(tacn)2	3	2	8.58	2	3	0.95
Ti(cdta)	0	1	8.80	-1	2	-0.02
Ti(edta)	0	1	8.24	-1	2	0.02
Ti(hbed)	0	1	9.28	-1	2	-0.64
Ti(nap)3	-2	1	10.00	-3	2	-1.12
V(edta)	-1	3	8.27	-2	4	-1.04

Supporting Information 4: How to obtain the redox potential

	A	B	C	D	E	F	G	H	I	J	K
1	How to compute the redox potential										
2	Parameters		Eps=	78.39							
3	Taken from the Table 2		a=	15.26	μ=	0.0383	ESHE=	4.26			
4			Prefactor	0.880205657	<= Obtained by "=27.211*(1-1/D2)/(2*D3)"						
5	Computed redox potential can be obtained by "=H9-(D4*(C9)^2*ERF(F3*ABS(C9)*D9)/D9-D4*(F9)^2*ERF(F3*ABS(F9)*D9)/D9+H3"										
6			Ox			Red					
7				(In a.u.)	(In a.u.)		(In a.u.)	Calc. in eV		Computed	Absolute Error
8	**Compound**	Cavity volume	Charge	Cavity radii	Optimized Energy	Charge	Optimized Energy	EAIP	Exp.		
9	Co(h2o)2(phen)2	526.081	3	9.463541901	−1441.953965	2	−1442.166538	5.78	0.68	1.05	0.37
10											
11		Taken from "Cavity Volume"		Obtained by "=((3*B9)/(4*PI())^(1/3)/0.529177"				Obtained by "=(E9-G9)*27.211"			

Figure S2: Screenshot of "Microsoft Excel" (example for B3LYP).

Supporting Information 5: Detailed data for DFT comparison.

Table S4: Result for BLYP.

**Compound**	Ox	Red	E_{AIP}	Comp.	Abs.Error
Co(cdta)	-1401.478472	-1401.600165	3.31	-0.13	0.48
Co(edta)	-1245.522163	-1245.645099	3.35	-0.08	0.45
Co(h2o)2(phen)2	-1441.141318	-1441.323904	4.97	0.73	0.05
Co(ox)2(h2o)2	-1053.149385	-1053.299776	4.09	0.71	0.07
Co(ox)3	-1277.745511	-1277.857509	3.05	0.11	0.46
Co(pdta)	-1284.811474	-1284.929443	3.21	-0.22	0.58
Cr(cdta)	-1342.680921	-1342.771417	2.46	-0.98	0.02
Cr(cl)(l15)	-1295.177825	-1295.300457	3.34	-0.74	0.11
Cr(CN)6	-644.4780183	-644.5630493	2.31	-0.61	0.67
Cr(co)5(pycn)	-994.1253158	-994.2320337	2.90	-0.76	0.27
Cr(cyclam)(cl)2	-1621.638139	-1621.748251	3.00	-0.86	0.11
Cr(cyclam)(cn)2	-886.8491526	-886.9516763	2.79	-1.07	0.05
Cr(edta)	-1186.725573	-1186.814329	2.42	-1.01	0.03
Cr(en)2(cn)2	-653.5393372	-653.6427896	2.82	-1.05	0.17
Cr(en)2(ncs)2	-1449.962906	-1450.064353	2.76	-1.10	0.20
Cr(en)3	-658.0426022	-658.169449	3.45	-0.88	0.15
Cr(mida)2	-1187.927565	-1188.011271	2.28	-1.15	0.01
Cr(tacn)2	-890.1120099	-890.235467	3.36	-0.93	0.21
Fe(bpy)(cn)4	-990.676262	-990.8302382	4.19	0.76	0.22
Fe(bpy)2(cn)2	-1299.953212	-1300.126709	4.72	0.87	0.09
Fe(cdta)	-1379.547319	-1379.680225	3.62	0.17	0.13
Fe(cl)(l52)	-1672.501605	-1672.678923	4.83	0.77	0.04
Fe(CN)6	-681.3849618	-681.521673	3.72	0.81	0.45
Fe(edta)	-1223.592859	-1223.726478	3.64	0.21	0.09
Fe(hbed)	-1458.83075	-1458.942695	3.05	-0.42	0.05
Fe(nap)3	-1729.381834	-1729.464574	2.25	-0.88	0.30
Fe(ox)3	-1255.825104	-1255.941392	3.16	0.21	0.20
Fe(py26diox)2	-1292.632496	-1292.7709	3.77	0.32	0.12
Fe(py2ox)	-1372.681093	-1372.826426	3.95	0.28	0.07
Mn(cn)5(no)	-698.729214	-698.8900232	4.38	1.23	0.63
Mn(CN)6	-661.805124	-661.9174721	3.06	0.15	0.39
Ni(edta)	-1270.588312	-1270.749228	4.38	0.96	0.33
Ni(pdtc)	-2711.234752	-2711.376159	3.85	0.40	0.02
Ni(tacn)2	-973.997903	-974.1933993	5.32	1.03	0.08
Ti(cdta)	-1313.919743	-1314.048937	3.52	-0.15	0.13
Ti(edta)	-1157.965346	-1158.094916	3.53	-0.14	0.16
Ti(hbed)	-1393.228612	-1393.330168	2.76	-0.91	0.27
Ti(nap)3	-1663.817878	-1663.890691	1.98	-1.33	0.21
V(edta)	-1171.428836	-1171.528483	2.71	-0.71	0.33

Table S5: Result for B3PW91.

**Compound**	Ox	Red	E_{AIP}	Comp.	Abs.Error
Co(cdta)	-1401.425856	-1401.58649	4.37	0.45	0.10
Co(edta)	-1245.421878	-1245.584168	4.42	0.50	0.13
Co(h2o)2(phen)2	-1441.157979	-1441.376886	5.96	1.20	0.52
Co(ox)2(h2o)2	-1052.958147	-1053.149712	5.21	1.30	0.52
Co(ox)3	-1277.470761	-1277.625021	4.20	1.03	0.46
Co(pdta)	-1284.724829	-1284.883385	4.31	0.40	0.04
Cr(cdta)	-1342.646814	-1342.753856	2.91	-1.01	0.05
Cr(cl)(l15)	-1295.354238	-1295.483417	3.52	-0.94	0.09
Cr(CN)6	-644.3964661	-644.4760339	2.17	-0.98	0.30
Cr(co)5(pycn)	-993.9555186	-994.0678724	3.06	-1.09	0.06
Cr(cyclam)(cl)2	-1621.759935	-1621.885232	3.41	-0.82	0.07
Cr(cyclam)(cn)2	-886.9869446	-887.0885465	2.76	-1.47	0.35
Cr(edta)	-1186.643775	-1186.748409	2.85	-1.07	0.09
Cr(en)2(cn)2	-653.6143941	-653.7317247	3.19	-1.04	0.18
Cr(en)2(ncs)2	-1449.970301	-1450.090829	3.28	-0.95	0.05
Cr(en)3	-658.1956512	-658.3391244	3.90	-0.91	0.12
Cr(mida)2	-1187.853533	-1187.952501	2.69	-1.23	0.07
Cr(tacn)2	-890.3140891	-890.4535376	3.79	-1.00	0.14
Fe(bpy)(cn)4	-990.6272823	-990.7811597	4.19	0.27	0.27
Fe(bpy)2(cn)2	-1299.951773	-1300.126808	4.76	0.53	0.25
Fe(cdta)	-1379.522929	-1379.670112	4.01	0.08	0.04
Fe(cl)(l52)	-1672.611104	-1672.804479	5.26	0.81	0.00
Fe(CN)6	-681.2889397	-681.4248346	3.70	0.56	0.20
Fe(edta)	-1223.520627	-1223.668865	4.03	0.12	0.00
Fe(hbed)	-1458.844728	-1458.972905	3.49	-0.44	0.07
Fe(nap)3	-1729.375649	-1729.472026	2.62	-0.76	0.18
Fe(ox)3	-1255.581721	-1255.712303	3.55	0.38	0.37
Fe(py26diox)2	-1292.496354	-1292.636832	3.82	-0.10	0.30
Fe(py2ox)	-1372.599925	-1372.74827	4.04	-0.11	0.46
Mn(cn)5(no)	-698.6002844	-698.7538461	4.18	0.65	0.05
Mn(CN)6	-661.7135777	-661.8237812	3.00	-0.14	0.10
Ni(edta)	-1270.493123	-1270.689075	5.33	1.42	0.13
Ni(pdtc)	-2711.032838	-2711.200862	4.57	0.65	0.27
Ni(tacn)2	-974.1883365	-974.4161935	6.20	1.40	0.45
Ti(cdta)	-1313.863236	-1314.011316	4.03	-0.12	0.10
Ti(edta)	-1157.861098	-1158.009545	4.04	-0.11	0.13
Ti(hbed)	-1393.212493	-1393.334047	3.31	-0.84	0.20
Ti(nap)3	-1663.782493	-1663.872524	2.45	-1.19	0.07
V(edta)	-1171.342363	-1171.455833	3.09	-0.83	0.21

Table S6: Result for BHandHLYP.

**Compound**	Ox	Red	E_{AIP}	Comp.	Abs.Error
Co(cdta)	-1400.995857	-1401.198457	5.51	1.19	0.84
Co(edta)	-1245.035706	-1245.240094	5.56	1.24	0.87
Co(h2o)2(phen)2	-1440.692839	-1440.950693	7.02	1.64	0.96
Co(ox)2(h2o)2	-1052.642208	-1052.871272	6.23	1.91	1.13
Co(ox)3	-1277.133341	-1277.326971	5.27	2.07	1.50
Co(pdta)	-1284.328018	-1284.527676	5.43	1.11	0.75
Cr(cdta)	-1342.296767	-1342.417084	3.27	-1.05	0.09
Cr(cl)(l15)	-1295.055578	-1295.196775	3.84	-1.08	0.23
Cr(CN)6	-644.1764159	-644.2432113	1.82	-1.37	0.09
Cr(co)5(pycn)	-993.6116701	-993.7139819	2.78	-1.80	0.77
Cr(cyclam)(cl)2	-1621.53501	-1621.673274	3.76	-0.90	0.15
Cr(cyclam)(cn)2	-886.6584326	-886.7552112	2.63	-2.03	0.91
Cr(edta)	-1186.337537	-1186.455201	3.20	-1.12	0.14
Cr(en)2(cn)2	-653.346382	-653.4744123	3.48	-1.18	0.04
Cr(en)2(ncs)2	-1449.764856	-1449.897332	3.60	-1.06	0.16
Cr(en)3	-657.9043593	-658.0592925	4.22	-1.18	0.15
Cr(mida)2	-1187.544448	-1187.653689	2.97	-1.35	0.19
Cr(tacn)2	-889.9663058	N/A	N/A	N/A	N/A
Fe(bpy)(cn)4	-990.270206	-990.4207104	4.10	-0.23	0.77
Fe(bpy)2(cn)2	-1299.525856	-1299.701981	4.79	0.13	0.65
Fe(cdta)	-1379.149662	-1379.302523	4.16	-0.17	0.21
Fe(cl)(l52)	-1672.246714	-1672.447832	5.47	0.55	0.26
Fe(CN)6	-681.0017617	-681.1289093	3.46	0.28	0.08
Fe(edta)	-1223.19106	-1223.345209	4.19	-0.13	0.25
Fe(hbed)	-1458.438424	-1458.570645	3.60	-0.73	0.36
Fe(nap)3	-1728.907959	-1729.003567	2.60	-0.71	0.13
Fe(ox)3	-1255.299055	-1255.434177	3.68	0.48	0.47
Fe(py26diox)2	N/A	-1292.186745	N/A	N/A	N/A
Fe(py2ox)	-1372.1307	-1372.288379	4.29	-0.29	0.64
Mn(cn)5(no)	-698.3315638	-698.4686458	3.73	-0.11	0.71
Mn(CN)6	-661.4572019	-661.555817	2.68	-0.50	0.26
Ni(edta)	-1270.085572	-1270.32119	6.41	2.09	0.80
Ni(pdtc)	-2710.754727	-2710.950588	5.33	1.00	0.62
Ni(tacn)2	-973.7411736	-974.0058379	7.20	1.81	0.86
Ti(cdta)	-1313.525239	-1313.698587	4.72	0.14	0.16
Ti(edta)	-1157.566782	-1157.740652	4.73	0.15	0.13
Ti(hbed)	-1392.844543	-1392.988787	3.93	-0.65	0.01
Ti(nap)3	-1663.355929	-1663.463946	2.94	-0.94	0.18
V(edta)	-1171.055002	-1171.175279	3.27	-1.05	0.01

a: N/A means that “could not be optimized” (SCF not converged). In this case, we removed the complex for the fitting set.

Table S7: Results for CAM-B3LYP

**Compound**	Ox	Red	E_{AIP}	Comp.	Abs.Error
Co(cdta)	-1401.290196	-1401.456247	4.52	0.35	0.00
Co(edta)	-1245.325044	-1245.492829	4.57	0.40	0.03
Co(h2o)2(phen)2	-1440.88894	-1441.11443	6.14	1.26	0.58
Co(ox)2(h2o)2	-1052.930632	-1053.125652	5.31	1.14	0.36
Co(ox)3	-1277.465967	-1277.625221	4.33	0.88	0.31
Co(pdta)	-1284.618123	-1284.782039	4.46	0.29	0.07
Cr(cdta)	-1342.526452	-1342.63877	3.06	-1.11	0.15
Cr(cl)(l15)	-1295.187668	-1295.321145	3.63	-0.95	0.10
Cr(CN)6	-644.3127353	-644.3903259	2.11	-1.33	0.05
Cr(co)5(pycn)	-993.8292745	-993.9384552	2.97	-1.38	0.35
Cr(cyclam)(cl)2	-1621.671283	-1621.801639	3.55	-0.86	0.11
Cr(cyclam)(cn)2	-886.7899801	-886.894265	2.84	-1.57	0.45
Cr(edta)	-1186.562188	-1186.671858	2.98	-1.18	0.20
Cr(en)2(cn)2	-653.4685038	-653.5889932	3.28	-1.13	0.09
Cr(en)2(ncs)2	-1449.906356	-1450.029996	3.36	-1.05	0.15
Cr(en)3	-658.0184918	-658.1650267	3.99	-0.92	0.11
Cr(mida)2	-1187.768154	-1187.869826	2.77	-1.40	0.24
Cr(tacn)2	-890.0913915	-890.2347693	3.90	-0.99	0.15
Fe(bpy)(cn)4	-990.4534345	-990.6083336	4.21	0.05	0.49
Fe(bpy)2(cn)2	-1299.70311	-1299.880745	4.83	0.42	0.36
Fe(cdta)	-1379.388886	-1379.544005	4.22	0.05	0.01
Fe(cl)(l52)	-1672.390979	-1672.591836	5.47	0.88	0.07
Fe(CN)6	-681.1903872	-681.3259865	3.69	0.26	0.10
Fe(edta)	-1223.425208	-1223.581651	4.26	0.09	0.03
Fe(hbed)	-1458.648802	-1458.782999	3.65	-0.52	0.15
Fe(nap)3	-1729.106031	-1729.205773	2.71	-0.86	0.28
Fe(ox)3	-1255.577224	-1255.716392	3.79	0.33	0.32
Fe(py26diox)2	-1292.312847	-1292.460093	4.01	-0.16	0.36
Fe(py2ox)	-1372.378146	-1372.534189	4.25	-0.10	0.45
Mn(cn)5(no)	-698.5103397	-698.6696278	4.33	0.49	0.11
Mn(CN)6	-661.6212131	-661.7294026	2.94	-0.49	0.25
Ni(edta)	-1270.38414	-1270.591981	5.66	1.49	0.20
Ni(pdtc)	-2711.027284	-2711.207855	4.91	0.74	0.36
Ni(tacn)2	-973.9406656	-974.1776793	6.45	1.56	0.61
Ti(cdta)	-1313.744371	-1313.904651	4.36	0.01	0.03
Ti(edta)	-1157.780934	-1157.938553	4.29	-0.06	0.08
Ti(hbed)	-1393.03434	-1393.165918	3.58	-0.77	0.13
Ti(nap)3	-1663.533444	-1663.631028	2.66	-1.23	0.11
V(edta)	-1171.268081	-1171.386065	3.21	-0.96	0.08

Table S8: Result for LC-wPBE

**Compound**	Ox	Red	E_{AIP}	Comp.	Abs.Error
Co(cdta)	-1401.117301	-1401.288018	4.65	0.48	0.13
Co(edta)	-1245.151897	-1245.323871	4.68	0.52	0.15
Co(h2o)2(phen)2	-1440.682146	-1440.911414	6.24	1.20	0.52
Co(ox)2(h2o)2	-1052.739498	-1052.943249	5.54	1.39	0.61
Co(ox)3	-1277.196101	-1277.363491	4.55	1.17	0.60
Co(pdta)	-1284.445039	-1284.614164	4.60	0.44	0.08
Cr(cdta)	-1342.341496	-1342.458111	3.17	-0.99	0.03
Cr(cl)(l15)	-1295.067023	-1295.205179	3.76	-0.96	0.11
Cr(CN)6	-644.1902802	-644.2657975	2.05	-1.31	0.03
Cr(co)5(pycn)	-993.6259523	-993.7384333	3.06	-1.34	0.31
Cr(cyclam)(cl)2	-1621.416743	-1621.551033	3.65	-0.83	0.08
Cr(cyclam)(cn)2	-886.7583482	-886.8615975	2.81	-1.68	0.56
Cr(edta)	-1186.376994	-1186.490487	3.09	-1.07	0.09
Cr(en)2(cn)2	-653.4428759	-653.5672352	3.38	-1.10	0.12
Cr(en)2(ncs)2	-1449.619135	-1449.750032	3.56	-0.92	0.02
Cr(en)3	-658.0411453	-658.1922649	4.11	-0.98	0.05
Cr(mida)2	-1187.58586	-1187.692207	2.89	-1.27	0.11
Cr(tacn)2	-890.1023594	-890.2504026	4.03	-1.04	0.10
Fe(bpy)(cn)4	-990.301854	-990.4571284	4.23	0.06	0.48
Fe(bpy)2(cn)2	-1299.506912	-1299.684769	4.84	0.35	0.43
Fe(cdta)	-1379.212891	-1379.373029	4.36	0.19	0.15
Fe(cl)(l52)	-1672.147689	-1672.354702	5.63	0.92	0.11
Fe(CN)6	-681.0833123	-681.220002	3.72	0.37	0.01
Fe(edta)	-1223.248763	-1223.410382	4.40	0.24	0.12
Fe(hbed)	-1458.449677	-1458.590156	3.82	-0.35	0.02
Fe(nap)3	-1728.807046	-1728.913372	2.89	-0.71	0.13
Fe(ox)3	-1255.30688	-1255.452176	3.95	0.56	0.55
Fe(py26diox)2	-1292.08984	-1292.237609	4.02	-0.15	0.35
Fe(py2ox)	-1372.155315	-1372.310741	4.23	-0.17	0.52
Mn(cn)5(no)	-698.3862437	-698.5432588	4.27	0.51	0.09
Mn(CN)6	-661.5054929	-661.6137301	2.95	-0.41	0.17
Ni(edta)	-1270.218207	-1270.429276	5.74	1.58	0.29
Ni(pdtc)	-2710.357179	-2710.54017	4.98	0.81	0.43
Ni(tacn)2	-973.9717651	-974.2110503	6.51	1.44	0.49
Ti(cdta)	-1313.545106	-1313.710054	4.49	0.09	0.11
Ti(edta)	-1157.581566	-1157.746729	4.49	0.10	0.08
Ti(hbed)	-1392.811493	-1392.948634	3.73	-0.67	0.03
Ti(nap)3	-1663.21014	-1663.313054	2.80	-1.07	0.05
V(edta)	-1171.076908	-1171.199747	3.34	-0.82	0.22

Table S9: Result for wB97X-D

**Compound**	Ox	Red	E_{AIP}	Comp.	Abs.Error
Co(cdta)	-1401.501533	-1401.673087	4.67	0.55	0.20
Co(edta)	-1245.477777	-1245.651152	4.72	0.60	0.23
Co(h2o)2(phen)2	-1441.19462	-1441.425061	6.27	1.39	0.71
Co(ox)2(h2o)2	-1052.990791	-1053.191808	5.47	1.35	0.57
Co(ox)3	-1277.519803	-1277.684641	4.49	1.20	0.63
Co(pdta)	-1284.78817	-1284.95716	4.60	0.48	0.12
Cr(cdta)	-1342.727589	-1342.837871	3.00	-1.12	0.16
Cr(cl)(l15)	-1295.429664	-1295.56027	3.55	-0.99	0.14
Cr(CN)6	-644.3955773	-644.4749293	2.16	-1.12	0.16
Cr(co)5(pycn)	-993.9584809	-994.0667254	2.95	-1.35	0.32
Cr(cyclam)(cl)2	-1621.856718	-1621.985702	3.51	-0.85	0.10
Cr(cyclam)(cn)2	-887.026375	-887.1286546	2.78	-1.58	0.46
Cr(edta)	-1186.704648	-1186.81272	2.94	-1.18	0.20
Cr(en)2(cn)2	-653.6214971	-653.7403939	3.24	-1.13	0.09
Cr(en)2(ncs)2	-1450.03471	-1450.156889	3.32	-1.04	0.14
Cr(en)3	-658.2086325	-658.3508689	3.87	-1.02	0.01
Cr(mida)2	-1187.913277	-1188.01147	2.67	-1.45	0.29
Cr(tacn)2	-890.3591479	-890.4983175	3.79	-1.10	0.04
Fe(bpy)(cn)4	-990.6279646	-990.7911985	4.44	0.32	0.22
Fe(bpy)2(cn)2	-1299.980285	-1300.159489	4.88	0.52	0.26
Fe(cdta)	-1379.60095	-1379.755948	4.22	0.10	0.06
Fe(cl)(l52)	-1672.693652	-1672.893412	5.44	0.89	0.08
Fe(CN)6	-681.2789813	-681.4159173	3.73	0.45	0.09
Fe(edta)	-1223.5785	-1223.734957	4.26	0.14	0.02
Fe(hbed)	-1458.909951	-1459.045201	3.68	-0.44	0.07
Fe(nap)3	-1729.406516	-1729.503391	2.64	-0.71	0.13
Fe(ox)3	-1255.630816	-1255.770232	3.79	0.51	0.50
Fe(py26diox)2	-1292.499169	-1292.64642	4.01	-0.11	0.31
Fe(py2ox)	-1372.617935	-1372.774456	4.26	-0.04	0.39
Mn(cn)5(no)	-698.5884486	-698.7477161	4.33	0.57	0.03
Mn(CN)6	-661.7070586	-661.8187703	3.04	-0.24	0.00
Ni(edta)	-1270.543496	-1270.755161	5.76	1.64	0.35
Ni(pdtc)	-2711.158304	-2711.340522	4.96	0.84	0.46
Ni(tacn)2	-974.2265249	-974.4650487	6.49	1.60	0.65
Ti(cdta)	-1313.951002	-1314.105126	4.19	-0.11	0.09
Ti(edta)	-1157.928828	-1158.086456	4.29	-0.01	0.03
Ti(hbed)	-1393.288793	-1393.41838	3.53	-0.77	0.13
Ti(nap)3	-1663.826186	-1663.921453	2.59	-1.19	0.07
V(edta)	-1171.410538	-1171.52575	3.14	-0.98	0.06

Table S10: Result for LC-BOP

**Compound**	Ox	Red	E_{AIP}	Comp.	Abs.Error
Co(cdta)	-1398.569648	-1398.731855	4.41	0.29	0.06
Co(edta)	-1243.005738	-1243.169476	4.46	0.34	0.03
Co(h2o)2(phen)2	-1437.646082	-1437.86952	6.08	1.05	0.37
Co(ox)2(h2o)2	-1050.43729	-1050.62629	5.14	1.03	0.25
Co(ox)3	-1274.539319	-1274.693061	4.18	0.88	0.31
Co(pdta)	-1281.453843	-1281.609922	4.25	0.13	0.23
Cr(cdta)	-1339.153147	-1339.272203	3.24	-0.89	0.07
Cr(cl)(l15)	-1293.004151	-1293.142013	3.75	-0.95	0.10
Cr(CN)6	-642.9203213	-642.9960812	2.06	-1.22	0.06
Cr(co)5(pycn)	-991.7608487	-991.8676113	2.91	-1.46	0.43
Cr(cyclam)(cl)2	-1619.500456	-1619.635928	3.69	-0.77	0.02
Cr(cyclam)(cn)2	-884.7932589	-884.8977638	2.84	-1.62	0.50
Cr(edta)	-1184.26271	-1184.375874	3.08	-1.04	0.06
Cr(en)2(cn)2	-652.0711733	-652.1944205	3.35	-1.11	0.11
Cr(en)2(ncs)2	-1447.93072	-1448.059691	3.51	-0.95	0.05
Cr(en)3	-656.6191454	-656.7693042	4.09	-1.01	0.02
Cr(mida)2	-1184.788256	-1184.896688	2.95	-1.17	0.01
Cr(tacn)2	-888.0990164	-888.2459699	4.00	-1.07	0.07
Fe(bpy)(cn)4	-988.2365772	-988.3991995	4.43	0.30	0.24
Fe(bpy)2(cn)2	-1296.68515	-1296.864897	4.89	0.43	0.35
Fe(cdta)	-1376.209102	-1376.364293	4.22	0.10	0.06
Fe(cl)(l52)	-1669.283082	-1669.49007	5.63	0.94	0.13
Fe(CN)6	-660.2223508	-660.3316807	2.97	-0.30	0.06
Fe(edta)	-1221.105768	-1221.267645	4.40	0.28	0.16
Fe(hbed)	-1455.555189	-1455.693774	3.77	-0.36	0.01
Fe(nap)3	-1725.215484	-1725.318145	2.79	-0.74	0.16
Fe(ox)3	-1252.9358	-1253.075427	3.80	0.49	0.48
Fe(py26diox)2	-1289.606534	-1289.757921	4.12	0.00	0.20
Fe(py2ox)	-1369.385934	-1369.546435	4.37	0.00	0.35
Mn(cn)5(no)	-696.6850302	-696.8402213	4.22	0.52	0.08
Mn(CN)6	-679.7880703	-679.9245366	3.71	0.45	0.09
Ni(edta)	-1268.051049	-1268.261433	5.72	1.60	0.31
Ni(pdtc)	-2705.99095	-2706.175567	5.02	0.90	0.52
Ni(tacn)2	-971.9169473	-972.154612	6.47	1.40	0.45
Ti(cdta)	-1311.059359	-1311.227029	4.56	0.19	0.21
Ti(edta)	-1155.497241	-1155.665118	4.57	0.20	0.18
Ti(hbed)	-1389.977873	-1390.115403	3.74	-0.63	0.01
Ti(nap)3	-1659.681685	-1659.784101	2.79	-1.03	0.09
V(edta)	-1168.978948	-1169.099051	3.27	-0.85	0.19

Table S11: Result for LC-BOP12

**Compound**	Ox	Red	E_{AIP}	Comp.	Abs.Error
Co(cdta)	-1397.978963	-1398.132483	4.18	0.20	0.15
Co(edta)	-1242.500124	-1242.655166	4.22	0.25	0.12
Co(h2o)2(phen)2	-1437.027737	-1437.24214	5.83	0.99	0.31
Co(ox)2(h2o)2	-1050.043765	-1050.224146	4.91	0.95	0.17
Co(ox)3	-1274.077565	-1274.222676	3.95	0.76	0.19
Co(pdta)	-1280.924943	-1281.072453	4.01	0.04	0.32
Cr(cdta)	-1338.575307	-1338.688556	3.08	-0.89	0.07
Cr(cl)(l15)	-1292.502848	-1292.635203	3.60	-0.93	0.08
Cr(CN)6	-642.6699935	-642.7439868	2.01	-1.15	0.13
Cr(co)5(pycn)	-991.3870437	-991.4917477	2.85	-1.36	0.33
Cr(cyclam)(cl)2	-1619.006199	-1619.13633	3.54	-0.76	0.01
Cr(cyclam)(cn)2	-884.3551896	-884.45617	2.75	-1.55	0.43
Cr(edta)	-1183.770547	-1183.878123	2.93	-1.04	0.06
Cr(en)2(cn)2	-651.7563798	-651.8745433	3.22	-1.08	0.14
Cr(en)2(ncs)2	-1447.505937	-1447.629297	3.36	-0.94	0.04
Cr(en)3	-656.2723535	-656.4166539	3.93	-0.98	0.05
Cr(mida)2	-1184.288038	-1184.390914	2.80	-1.17	0.01
Cr(tacn)2	-887.6368353	-887.7779767	3.84	-1.04	0.10
Fe(bpy)(cn)4	-987.8299255	-987.9897492	4.35	0.38	0.16
Fe(bpy)2(cn)2	-1296.13006	-1296.306001	4.79	0.49	0.29
Fe(cdta)	-1375.620678	-1375.770239	4.07	0.10	0.06
Fe(cl)(l52)	-1668.65899	-1668.859445	5.45	0.93	0.12
Fe(CN)6	-659.9684533	-660.0758328	2.92	-0.24	0.00
Fe(edta)	-1220.603248	-1220.758868	4.23	0.27	0.15
Fe(hbed)	-1454.923902	-1455.056503	3.61	-0.37	0.00
Fe(nap)3	-1724.481455	-1724.57908	2.66	-0.76	0.18
Fe(ox)3	-1252.476207	-1252.610561	3.66	0.46	0.45
Fe(py26diox)2	-1289.093253	-1289.240618	4.01	0.04	0.16
Fe(py2ox)	-1368.819576	-1368.975384	4.24	0.03	0.32
Mn(cn)5(no)	-696.424472	-696.5794536	4.22	0.65	0.05
Mn(CN)6	-679.5296063	-679.6645485	3.67	0.52	0.16
Ni(edta)	-1267.539853	-1267.74138	5.48	1.52	0.23
Ni(pdtc)	-2705.335738	-2705.512693	4.82	0.84	0.46
Ni(tacn)2	-971.4354289	-971.6641716	6.22	1.34	0.39
Ti(cdta)	-1310.496862	-1310.656514	4.34	0.14	0.16
Ti(edta)	-1155.019812	-1155.179666	4.35	0.14	0.12
Ti(hbed)	-1389.371193	-1389.501194	3.54	-0.67	0.03
Ti(nap)3	-1658.971547	-1659.067216	2.60	-1.08	0.04
V(edta)	-1168.49042	-1168.60618	3.15	-0.82	0.22

Table S12: Result for LCgau-BOP

**Compound**	Ox	Red	E_{AIP}	Comp.	Abs.Error
Co(cdta)	-1402.732764	-1402.881637	4.05	0.10	0.25
Co(edta)	-1246.654761	-1246.80514	4.09	0.15	0.22
Co(h2o)2(phen)2	-1441.953965	-1442.166538	5.78	1.02	0.34
Co(ox)2(h2o)2	-1053.42855	-1053.605272	4.81	0.87	0.09
Co(ox)3	-1278.332683	-1278.473535	3.83	0.68	0.11
Co(pdta)	-1285.228143	-1285.371893	3.91	-0.04	0.40
Cr(cdta)	-1343.436174	-1343.54275	2.90	-1.05	0.09
Cr(cl)(l15)	-1295.518504	-1295.645209	3.45	-1.00	0.15
Cr(CN)6	-644.920808	-644.9972568	2.08	-1.06	0.22
Cr(co)5(pycn)	-994.9248481	-995.0296923	2.85	-1.31	0.28
Cr(cyclam)(cl)2	-1621.572131	-1621.696561	3.39	-0.85	0.10
Cr(cyclam)(cn)2	-887.5164111	-887.6194086	2.80	-1.43	0.31
Cr(edta)	-1188.033111	-1188.133925	2.74	-1.20	0.22
Cr(en)2(cn)2	-654.0202026	-654.1332121	3.08	-1.16	0.06
Cr(en)2(ncs)2	-1449.952976	-1450.071337	3.22	-1.01	0.11
Cr(en)3	-658.5424999	-658.6812409	3.78	-1.03	0.00
Cr(mida)2	-1188.553338	-1188.649571	2.62	-1.33	0.17
Cr(tacn)2	-890.8002683	-890.9357243	3.69	-1.10	0.04
Fe(bpy)(cn)4	-991.2001928	-991.3615288	4.39	0.44	0.10
Fe(bpy)2(cn)2	-1300.683798	-1300.860393	4.81	0.57	0.21
Fe(cdta)	-1380.413976	-1380.562122	4.03	0.08	0.04
Fe(cl)(l52)	-1672.922993	-1673.121051	5.39	0.95	0.14
Fe(CN)6	-662.1930721	-662.302535	2.98	-0.15	0.09
Fe(edta)	-1224.798759	-1224.951442	4.15	0.21	0.09
Fe(hbed)	-1460.021944	-1460.152288	3.55	-0.40	0.03
Fe(nap)3	-1730.595477	-1730.689838	2.57	-0.76	0.18
Fe(ox)3	-1256.773222	-1256.905746	3.61	0.45	0.44
Fe(py26diox)2	-1293.619854	-1293.768389	4.04	0.09	0.11
Fe(py2ox)	-1373.651893	-1373.809016	4.28	0.12	0.23
Mn(cn)5(no)	-698.7915476	-698.9470159	4.23	0.66	0.06
Mn(CN)6	-681.7180507	-681.85326	3.68	0.55	0.19
Ni(edta)	-1271.634517	-1271.836415	5.49	1.55	0.26
Ni(pdtc)	-2709.211042	-2709.391473	4.91	0.96	0.58
Ni(tacn)2	-974.4302714	-974.6604419	6.26	1.47	0.52
Ti(cdta)	-1315.388196	-1315.547505	4.33	0.18	0.20
Ti(edta)	-1159.3119	-1159.471521	4.34	0.18	0.16
Ti(hbed)	-1394.565137	-1394.695699	3.55	-0.61	0.03
Ti(nap)3	-1665.181185	-1665.277107	2.61	-1.03	0.09
V(edta)	-1172.771613	-1172.884902	3.08	-0.86	0.18

Table S13: Result for LCgau-BOP12

**Compound**	Ox	Red	E_{AIP}	Comp.	Abs.Error
Co(cdta)	-1399.082529	-1399.245802	4.44	0.41	0.06
Co(edta)	-1243.384335	-1243.549126	4.48	0.45	0.08
Co(h2o)2(phen)2	-1438.560719	-1438.786425	6.14	1.24	0.56
Co(ox)2(h2o)2	-1050.568262	-1050.757821	5.16	1.13	0.35
Co(ox)3	-1274.772033	-1274.92632	4.20	1.07	0.50
Co(pdta)	-1281.861448	-1282.018806	4.28	0.25	0.11
Cr(cdta)	-1339.69804	-1339.810125	3.05	-0.99	0.03
Cr(cl)(l15)	-1293.477579	-1293.609425	3.59	-0.95	0.10
Cr(CN)6	-643.2796625	-643.3520353	1.97	-1.15	0.13
Cr(co)5(pycn)	-992.2308672	-992.3358382	2.86	-1.39	0.36
Cr(cyclam)(cl)2	-1619.89213	-1620.021672	3.52	-0.80	0.05
Cr(cyclam)(cn)2	-885.304398	-885.4040416	2.71	-1.61	0.49
Cr(edta)	-1184.67545	-1184.781962	2.90	-1.14	0.16
Cr(en)2(cn)2	-652.3774486	-652.495063	3.20	-1.12	0.10
Cr(en)2(ncs)2	-1448.24465	-1448.36751	3.34	-0.98	0.08
Cr(en)3	-656.8992806	-657.0428911	3.91	-1.03	0.00
Cr(mida)2	-1185.192952	-1185.294612	2.77	-1.27	0.11
Cr(tacn)2	-888.5899268	-888.7308678	3.84	-1.09	0.05
Fe(bpy)(cn)4	-988.8547846	-989.014107	4.34	0.30	0.24
Fe(bpy)2(cn)2	-1297.589227	-1297.764662	4.77	0.45	0.33
Fe(cdta)	-1376.739987	-1376.885588	3.96	-0.07	0.11
Fe(cl)(l52)	-1670.10715	-1670.304323	5.37	0.83	0.02
Fe(CN)6	-660.5689867	-660.6749832	2.88	-0.23	0.01
Fe(edta)	-1221.505057	-1221.656562	4.12	0.09	0.03
Fe(hbed)	-1456.300448	-1456.429444	3.51	-0.53	0.16
Fe(nap)3	-1726.295262	-1726.388369	2.53	-0.73	0.15
Fe(ox)3	-1253.189333	-1253.319095	3.53	0.40	0.39
Fe(py26diox)2	-1290.249828	-1290.396825	4.00	-0.04	0.24
Fe(py2ox)	-1370.161666	-1370.317643	4.24	-0.01	0.36
Mn(cn)5(no)	-697.0030734	-697.1558373	4.16	0.53	0.07
Mn(CN)6	-680.1216674	-680.2545073	3.61	0.50	0.14
Ni(edta)	-1268.424454	-1268.633857	5.70	1.66	0.37
Ni(pdtc)	-2706.52348	-2706.708736	5.04	1.01	0.63
Ni(tacn)2	-972.3671619	-972.6045829	6.46	1.54	0.59
Ti(cdta)	-1311.628658	-1311.78934	4.37	0.12	0.14
Ti(edta)	-1155.932194	-1156.093132	4.38	0.13	0.11
Ti(hbed)	-1390.758088	-1390.889567	3.58	-0.67	0.03
Ti(nap)3	-1660.795128	-1660.891962	2.63	-1.04	0.08
V(edta)	-1169.39943	-1169.514613	3.13	-0.90	0.14

Table S14: Result for LCgau-B97

**Compound**	Ox	Red	E_{AIP}	Comp.	Abs.Error
Co(cdta)	-1402.753375	-1402.928218	4.76	0.60	0.25
Co(edta)	-1246.586686	-1246.76306	4.80	0.65	0.28
Co(h2o)2(phen)2	-1442.499658	-1442.735378	6.41	1.37	0.69
Co(ox)2(h2o)2	-1053.181617	-1053.379306	5.38	1.24	0.46
Co(ox)3	-1277.921071	-1278.082862	4.40	1.04	0.47
Co(pdta)	-1285.191343	-1285.357556	4.52	0.37	0.01
Cr(cdta)	-1343.272046	-1343.390219	3.22	-0.94	0.02
Cr(cl)(l15)	-1296.321389	-1296.458789	3.74	-0.98	0.13
Cr(CN)6	-644.9699716	-645.0498834	2.17	-1.17	0.11
Cr(co)5(pycn)	-994.8575328	-994.9654033	2.94	-1.46	0.43
Cr(cyclam)(cl)2	-1622.773938	-1622.907745	3.64	-0.84	0.09
Cr(cyclam)(cn)2	-887.8207107	-887.9271633	2.90	-1.59	0.47
Cr(edta)	-1187.777977	-1187.892522	3.12	-1.03	0.05
Cr(en)2(cn)2	-654.203005	-654.3277454	3.39	-1.09	0.13
Cr(en)2(ncs)2	-1450.889662	-1451.01888	3.52	-0.97	0.07
Cr(en)3	-658.7919071	-658.9425602	4.10	-1.00	0.03
Cr(mida)2	-1188.248104	N/A	N/A	N/A	N/A
Cr(tacn)2	-891.1515534	-891.299481	4.03	-1.05	0.09
Fe(bpy)(cn)4	-991.5193902	-991.6825829	4.44	0.29	0.25
Fe(bpy)2(cn)2	-1301.161783	-1301.342221	4.91	0.43	0.35
Fe(cdta)	-1380.37721	-1380.526319	4.06	-0.10	0.14
Fe(cl)(l52)	-1673.929693	-1674.136118	5.62	0.90	0.09
Fe(CN)6	-662.2924053	-662.4043526	3.05	-0.29	0.05
Fe(edta)	-1224.672522	-1224.835117	4.42	0.27	0.15
Fe(hbed)	-1460.229094	-1460.370857	3.86	-0.30	0.07
Fe(nap)3	-1731.000886	-1731.109439	2.95	-0.64	0.06
Fe(ox)3	-1256.297134	-1256.43801	3.83	0.46	0.45
Fe(py26diox)2	-1293.671731	-1293.820042	4.04	-0.12	0.32
Fe(py2ox)	-1373.86583	-1374.023447	4.29	-0.10	0.45
Mn(cn)5(no)	-698.805913	-698.9639036	4.30	0.55	0.05
Mn(CN)6	-681.8765089	-682.0133296	3.72	0.40	0.04
Ni(edta)	-1271.666529	-1271.879107	5.78	1.63	0.34
Ni(pdtc)	-2711.078521	-2711.260443	4.95	0.79	0.41
Ni(tacn)2	-975.0673054	-975.3079552	6.55	1.47	0.52
Ti(cdta)	-1315.146492	-1315.305114	4.32	-0.08	0.06
Ti(edta)	-1158.981434	-1159.139851	4.31	-0.08	0.10
Ti(hbed)	-1394.566399	-1394.700709	3.65	-0.74	0.10
Ti(nap)3	-1665.37905	-1665.48019	2.75	-1.11	0.01
V(edta)	-1172.475857	-1172.595118	3.25	-0.91	0.13

a: N/A means that “could not be optimized” (SCF not converged). In this case, we removed the complex for the fitting set.

Supporting Information 6: The optimized geometries for heme compounds. (Corresponds to section 3.3.1)

```
* Sample Input for Theta=45.0 at B3LYP/6-31++G** level *
----
#p opt=modredundant ub3lyp/genecp scrf=(cpcm,solvent=water)
int=grid=ultrafine

HisFlip/Ox

-1 2
 Fe      0.87528200   -0.02740900    0.12894900
 C       4.23393800   -0.08751500   -0.40354200
 H       5.29943500   -0.10648900   -0.59587600
 N       2.27025400   -1.49196600    0.03184100
 C       3.61043100   -1.31162000   -0.19435500
 C       2.08742100   -2.84853600    0.17676000
 C       4.30175400   -2.59367700   -0.20270600
 C       3.34063000   -3.55466200    0.04000800
 C       0.86019500   -3.46025400    0.42176400
 H       0.84808400   -4.54004000    0.51119400
 N      -0.52563400   -1.43675400    0.45510600
 C      -0.35405800   -2.80037100    0.54608300
 C      -1.86810500   -1.21664800    0.63638500
 C      -1.62155600   -3.45982200    0.78985300
 C      -2.57316800   -2.47337900    0.84616200
 C      -2.48436400    0.02929200    0.63334200
 H      -3.55543900    0.04708200    0.79539400
 N      -0.51201600    1.42900000    0.22611100
 C      -1.85693800    1.25347700    0.43915600
 C      -0.32832700    2.78659100    0.09092400
 C      -2.54982400    2.53431400    0.44076000
 C      -1.58925900    3.48912200    0.22270900
 C       0.89142700    3.40541900   -0.14710500
 H       0.88618600    4.48400500   -0.24825000
 N       2.28121600    1.39050000   -0.20060300
 C       2.11178800    2.74885900   -0.27978100
 C       3.62286400    1.16117000   -0.40322000
 C       3.38011500    3.40794400   -0.54804800
 C       4.32817100    2.40693900   -0.61776100
 N       0.57457000   -0.19394200   -1.85223900
 C       0.46531300   -1.33064854   -2.52967360
 C       0.45008039    0.83085265   -2.77695770
 N       0.27739779   -1.06924009   -3.83870670
```

C	0.26313488	0.30400511	-4.02779649
H	0.51769665	-2.32408185	-2.11474501
H	0.50420323	1.86648418	-2.48336448
H	0.16890068	-1.76950713	-4.56105922
C	-4.05682500	-2.61797700	1.02731300
H	-4.27223000	-3.53415800	1.58816800
H	-4.45512900	-1.80106700	1.63866200
C	-4.03162200	2.72252100	0.59502700
H	-4.24207100	3.71795800	0.99881600
H	-4.43264400	2.01681000	1.33184200
C	-4.80779900	2.56102100	-0.72593100
H	-4.40309800	3.25550900	-1.47643500
H	-4.64746100	1.55711600	-1.14186800
C	-4.83299400	-2.65945200	-0.30276900
H	-4.47705800	-3.49604900	-0.91910700
H	-4.61767000	-1.75358100	-0.88809400
C	-6.38038200	-2.78034400	-0.15690600
C	-6.34352900	2.80834500	-0.62096100
O	-6.86112400	-2.69638100	1.00542300
O	-6.99643600	2.56300800	-1.67164600
O	-6.78830800	3.23483800	0.47882500
O	-7.00719100	-2.94210600	-1.23909000
C	-1.80122400	-4.94191500	0.93498900
H	-2.83020500	-5.18772300	1.21134100
H	-1.57563500	-5.47129700	0.00018500
H	-1.14102000	-5.35734500	1.70633100
C	-1.75565100	4.97639400	0.12151200
H	-1.55003800	5.33989600	-0.89376000
H	-2.77609900	5.27518000	0.37656100
H	-1.07424200	5.50804900	0.79692500
C	3.51938700	-5.03556100	0.18413900
H	2.78886800	-5.46634100	0.87586300
H	3.40763900	-5.55887200	-0.77503800
H	4.51996000	-5.26795700	0.56287900
C	5.80576000	2.54188700	-0.82704500
H	6.36307800	1.75665700	-0.30694400
H	6.07726800	2.48451100	-1.88978200
H	6.16026700	3.50888200	-0.45601600
C	5.74107900	-2.74887600	-0.39653300
C	6.37061500	-3.81982000	-0.90705900
H	6.35516400	-1.90150900	-0.09440000
H	7.45333300	-3.83604500	-0.99257300
H	5.84130200	-4.69409700	-1.27246800
C	3.54124300	4.85482700	-0.66962900

```
 C                  4.47836300     5.50094200    -1.38224300
 H                  2.81812500     5.45963000    -0.12368600
 H                  4.51660800     6.58645500    -1.39008600
 H                  5.21456700     4.98449400    -1.99010100
 C                  0.07487745     0.94293328    -5.36374534
 H                  0.86314865     0.64643010    -6.06581169
 H                 -0.88851065     0.66783489    -5.80909701
 H                  0.10314113     2.03063791    -5.26133872
 N                  1.18584700     0.13851800     2.11008500
 C                  1.30887100    -0.86971300     2.96526500
 C                  1.31158600     1.30283400     2.85132200
 H                  1.26004100    -1.91841000     2.72098700
 C                  1.51327800     0.99132700     4.17021200
 H                  1.24836500     2.27532600     2.39098700
 H                  1.63113300    -0.96480500     5.03752700
 N                  1.50687200    -0.39429900     4.21109700
 C                  1.70765500     1.84361300     5.38028900
 H                  0.92494900     1.66599200     6.12724900
 H                  2.67483300     1.64849200     5.85853400
 H                  1.67487900     2.89916000     5.09897600

D 25 1 86 88 45.0 F

Fe 0
SDD
****
O,N,C,H 0
6-31++G(d,p)
****

Fe 0
SDD

----
```

Note: In this supporting material, we show an input for “B3LYP” level calculation. Without the modified Gaussian, no one can check the actual computed value discussed in the original paper. On the other hand, the results of LC-BOP12 are similar to those of B3LYP. Use these inputs for the check of reproductivity. Many readers will reproduce the input files by combining this supproting information.

Supporting Information 7: The optimized geometries for heme compounds.
(Corresponds to section 3.3.2)
Optimized by LC-BOP12/6-31++G(d,p) and SDD (for Fe)

Heme a (Ox, Protonated)

C	1.15796200	1.37393300	-3.46477200
N	1.50422300	0.05883900	-3.69390600
C	1.13806000	1.50201200	-2.11278300
C	1.67844300	-0.55364100	-2.51674900
N	1.46468600	0.29344000	-1.53070400
C	2.01389600	-1.60000800	4.24777900
N	1.69754500	-0.27685600	4.47336800
C	2.02024400	-1.73461000	2.89633200
C	1.52697500	0.33383200	3.29468700
N	1.71580100	-0.52185900	2.31092600
Fe	1.56817200	-0.11910500	0.39025800
C	4.83495000	0.76302300	0.37701200
C	2.40251700	-3.36170100	-0.33379300
C	-1.69537000	-0.97005600	0.39256400
C	0.71054500	3.11493300	1.12430100
N	3.28461100	-1.12015800	0.07768200
C	4.54401200	-0.57692400	0.11770200
C	5.52879900	-1.56538700	-0.14287200
C	4.83507700	-2.74143800	-0.35102100
C	3.44815400	-2.43174800	-0.20390900
C	5.42862600	-4.03672900	-0.66042000
O	4.81983900	-5.07395300	-0.86015400
C	7.00857100	-1.33593600	-0.21729300
C	7.45402200	-0.90584300	-1.61384500
C	8.93823400	-0.67385200	-1.68919200
O	9.32594800	-0.31027400	-2.91792200
O	9.71547500	-0.79233400	-0.76990700
N	0.56725000	-1.81227100	0.09543700
C	1.08607400	-3.06131800	-0.19378000
C	0.01524100	-4.03360200	-0.33866800
C	-1.13820700	-3.35536500	-0.15281400
C	-0.76997600	-1.97266700	0.12504600
C	0.24822300	-5.48292800	-0.62168200
N	-0.12335000	0.86741400	0.70456300
C	-1.37522400	0.33562100	0.66496600
C	-2.38057800	1.35419800	0.95490700
C	-1.70672300	2.50772500	1.15592300
C	-0.28874000	2.19541800	1.00286000
C	-3.84823800	1.09433800	1.03423800

C	-2.21949400	3.83994000	1.49457000
C	-3.30510200	4.39251200	0.96191900
N	2.56327700	1.59641900	0.68206600
C	2.06919300	2.81473600	0.96607800
C	3.12029200	3.79658600	1.09686000
C	4.28554500	3.13454300	0.88717200
C	3.93300300	1.75532200	0.62770400
C	2.89790000	5.24384500	1.39943500
C	5.67600300	3.69116000	0.87335600
C	6.12829200	4.07387600	-0.53435100
C	7.52570600	4.62912100	-0.55533000
O	8.24126500	4.77163900	0.40964600
O	7.91357700	4.96255800	-1.79313900
C	-2.55337400	-3.86059700	-0.16181800
O	-2.66194800	-4.88810100	-1.13777400
C	-2.96787000	-4.35613500	1.22616500
C	-4.44024000	-4.77432000	1.31674100
C	-5.37407600	-3.61642000	1.10011600
C	-6.27796300	-3.43704200	0.13539400
C	-7.09576600	-2.16748300	0.08760200
C	-6.82886300	-1.30803300	-1.15883100
C	-7.56206800	0.00145300	-1.09432000
C	-8.54985500	0.44595900	-1.87271700
C	-9.17052300	1.79917100	-1.60947800
C	-10.63342500	1.73046800	-1.14239800
C	-11.21025000	3.09883200	-0.91662000
C	-11.66361700	3.63623700	0.21612600
C	-12.20101600	5.04357400	0.24082700
C	-11.69993600	2.94233100	1.55122900
C	-6.56736600	-4.42750300	-0.96291900
C	-9.12985600	-0.31065700	-3.03896000
H	1.60969800	-0.38295900	-4.60133500
H	0.90981900	2.37679000	-1.52000700
H	1.95142200	-1.59511000	-2.41244300
H	1.60911600	0.17125400	5.37951800
H	2.22099800	-2.61780900	2.30629500
H	1.26941000	1.37900600	3.18794600
H	5.88536700	1.04017200	0.38193700
H	2.69094500	-4.38145600	-0.56417500
H	-2.74700800	-1.23301000	0.39032200
H	0.44366800	4.14310700	1.34660700
H	6.53544900	-4.04250800	-0.71157300
H	7.31396100	-0.57527800	0.50923100
H	7.54955200	-2.24435500	0.06713000

H	6.95008700	0.01774000	-1.92641600
H	7.18971100	-1.66128500	-2.36453800
H	-0.69985600	-6.01724700	-0.69117400
H	0.78763000	-5.61726500	-1.56767600
H	0.85736600	-5.94017200	0.16782000
H	-4.35660500	1.91493800	1.54915300
H	-4.29462400	1.00040600	0.03565700
H	-4.06058700	0.16659800	1.57788100
H	-1.65239900	4.40271500	2.23762000
H	-3.89013900	3.89271800	0.19169700
H	-3.63909700	5.38163900	1.26905000
H	2.33871200	5.37798900	2.33362800
H	2.32730600	5.73685400	0.60235400
H	3.84789700	5.77680100	1.50239800
H	5.72716700	4.57551700	1.51754800
H	6.38533400	2.97225200	1.29897700
H	5.45856600	4.82504100	-0.97207800
H	6.09677500	3.21184900	-1.21303100
H	-3.22087300	-3.03629100	-0.45076000
H	-3.60370600	-5.06990500	-1.28019700
H	-2.77094500	-3.55805900	1.95563100
H	-2.32089100	-5.20147800	1.49511300
H	-4.63390100	-5.59420100	0.61538900
H	-4.61236200	-5.19192900	2.31870500
H	-5.26406000	-2.81486800	1.83655100
H	-8.16690100	-2.41899100	0.11195800
H	-6.89288200	-1.56675700	0.98419300
H	-7.09438100	-1.86843600	-2.06116100
H	-5.74714400	-1.11366800	-1.21830000
H	-7.23911400	0.65397900	-0.27770600
H	-9.12374800	2.40637900	-2.52693000
H	-8.58479900	2.33241800	-0.84968600
H	-11.23422600	1.21720300	-1.90680200
H	-10.68957900	1.11347200	-0.23848100
H	-11.24035200	3.72497200	-1.81307400
H	-11.63109200	5.66831600	0.94266500
H	-13.24483900	5.05855500	0.58430100
H	-12.15992700	5.51468700	-0.74741500
H	-11.13567600	3.51973000	2.29636600
H	-12.73236400	2.87855700	1.92174600
H	-11.28777900	1.93022900	1.52733500
H	-6.18642400	-4.07166100	-1.92975800
H	-6.13703500	-5.41556900	-0.77530100
H	-7.65077300	-4.55836300	-1.08315800

H	-10.16682900	-0.61092900	-2.83961800
H	-8.56804500	-1.21529100	-3.28544800
H	-9.15310400	0.32627300	-3.93351700
C	2.27119300	-2.57212000	5.34609600
H	3.11316700	-2.25250800	5.97131200
H	1.39321200	-2.68451200	5.99300100
H	2.51208100	-3.55236200	4.92516200
C	0.88891500	2.34542100	-4.56089200
H	1.77002500	2.48048500	-5.19915100
H	0.06004500	2.01009600	-5.19530300
H	0.62239400	3.31800900	-4.13765500
H	10.29379600	-0.17668200	-2.89515800
H	8.82364200	5.31350800	-1.73276700

Heme a (Red, Protonated)

C	1.20541100	1.34323500	-3.58328000
N	1.54106900	0.02579500	-3.79701700
C	1.17551800	1.48030200	-2.22858600
C	1.69756700	-0.57068100	-2.59671000
N	1.48379900	0.28173200	-1.62354900
C	1.93157200	-1.55221700	4.33351700
N	1.59032900	-0.23500100	4.53943600
C	1.97591000	-1.69339200	2.97964000
C	1.44472000	0.35736400	3.33570400
N	1.67130200	-0.49754400	2.36755200
Fe	1.57805500	-0.10872500	0.37179400
C	4.85905200	0.76195200	0.40194000
C	2.39756100	-3.34767000	-0.30723100
C	-1.70989900	-0.94936400	0.34754000
C	0.72436600	3.12237900	1.05394100
N	3.30847400	-1.11755300	0.09659300
C	4.56919900	-0.58767100	0.14788300
C	5.55257900	-1.58644500	-0.09188000
C	4.85144600	-2.75987200	-0.30141800
C	3.45759100	-2.42957600	-0.17317500
C	5.44175500	-4.05237900	-0.59166800
O	4.84158100	-5.09800500	-0.79468900
C	7.03523600	-1.36622700	-0.15050400
C	7.49857800	-0.93614900	-1.54102600
C	8.98304400	-0.70547600	-1.60616500
O	9.38087800	-0.34230200	-2.83280700
O	9.75596100	-0.82348600	-0.68285000
N	0.54740400	-1.80938600	0.08292600
C	1.07178200	-3.05346600	-0.18809400

C	0.00264900	-4.03658000	-0.33796300
C	-1.15359600	-3.35944500	-0.16914000
C	-0.78489900	-1.96726900	0.10046500
C	0.23794000	-5.48957200	-0.60681800
N	-0.14334400	0.88919900	0.64955800
C	-1.39412000	0.36577400	0.60433300
C	-2.40088100	1.39972200	0.86724000
C	-1.71817800	2.54964600	1.05883700
C	-0.29459900	2.21338900	0.92712200
C	-3.87282000	1.15513500	0.93425200
C	-2.22168200	3.89044500	1.37344600
C	-3.31621300	4.44250600	0.85644600
N	2.59019100	1.60619300	0.65914600
C	2.08846900	2.82056000	0.92428000
C	3.14496700	3.81302000	1.06862900
C	4.31174600	3.15293600	0.88614100
C	3.95937500	1.76362200	0.62895600
C	2.91592700	5.26293000	1.35562100
C	5.70439200	3.70599200	0.89147200
C	6.19748800	4.03972300	-0.51485500
C	7.60041100	4.58004800	-0.52461500
O	8.30198700	4.74154400	0.44792600
O	8.01549100	4.87707000	-1.76386600
C	-2.56751700	-3.86619300	-0.18327500
O	-2.68241900	-4.89155900	-1.16436300
C	-2.98718300	-4.36796900	1.20086800
C	-4.45921800	-4.78820400	1.29045500
C	-5.39502700	-3.63019600	1.08374000
C	-6.30088700	-3.44411100	0.12225900
C	-7.11988700	-2.17490800	0.08591400
C	-6.85236300	-1.30255400	-1.15143000
C	-7.58749200	0.00517000	-1.07438100
C	-8.57849200	0.45422000	-1.84606200
C	-9.20146800	1.80365900	-1.56922900
C	-10.66165800	1.72696300	-1.09490600
C	-11.24143500	3.09172400	-0.85530700
C	-11.68997900	3.61895400	0.28412300
C	-12.23155400	5.02438000	0.32279800
C	-11.71649400	2.91466800	1.61401100
C	-6.59049400	-4.42583700	-0.98386700
C	-9.16042800	-0.29383500	-3.01685300
H	1.65137900	-0.42605900	-4.69736600
H	0.95075900	2.36272500	-1.64331800
H	1.96130500	-1.61342100	-2.47420500

H	1.46896100	0.21923300	5.43714500
H	2.20627900	-2.57787200	2.39980000
H	1.17589100	1.39814300	3.20700500
H	5.91201100	1.03445000	0.42112500
H	2.68384700	-4.37164000	-0.52603600
H	-2.76348000	-1.21086900	0.33897700
H	0.46256000	4.15615500	1.26197600
H	6.55073300	-4.05965900	-0.62667100
H	7.33611700	-0.60524300	0.57822600
H	7.57185700	-2.27634400	0.13804400
H	6.99809800	-0.01185300	-1.85733500
H	7.23917900	-1.69072600	-2.29449800
H	-0.70920000	-6.02440000	-0.69219300
H	0.79731400	-5.63424200	-1.54016000
H	0.83061400	-5.94572100	0.19648700
H	-4.37889600	1.97422300	1.45505800
H	-4.31681100	1.07622100	-0.06724000
H	-4.09860900	0.22233000	1.46427100
H	-1.63547100	4.46915900	2.08971200
H	-3.92358100	3.93328000	0.11003200
H	-3.63594300	5.43997000	1.15242100
H	2.34149400	5.40494200	2.27997500
H	2.35323100	5.74885600	0.54792700
H	3.86227500	5.80174900	1.46731700
H	5.74547700	4.61170200	1.50680900
H	6.39941000	2.99720800	1.35727400
H	5.54566500	4.78221200	-0.99284200
H	6.17180600	3.15603600	-1.16549300
H	-3.23506000	-3.04022000	-0.46837600
H	-3.62534100	-5.07624100	-1.29448100
H	-2.79140600	-3.57129900	1.93219000
H	-2.33990600	-5.21354100	1.46885200
H	-4.65354900	-5.60313500	0.58332700
H	-4.63088600	-5.21334000	2.28950800
H	-5.28443700	-2.83390000	1.82579700
H	-8.19094200	-2.42726900	0.10651400
H	-6.91854000	-1.58284400	0.98861500
H	-7.11579700	-1.85418200	-2.05980500
H	-5.77090900	-1.10594900	-1.20719300
H	-7.26351200	0.65140100	-0.25318800
H	-9.16128500	2.41816100	-2.48212300
H	-8.61329000	2.33263900	-0.80835200
H	-11.26501100	1.21781600	-1.86006500
H	-10.71103900	1.10273700	-0.19556300

H	-11.27854400	3.72469600	-1.74668800
H	-11.65953900	5.64544500	1.02619900
H	-13.27343800	5.03344900	0.67233000
H	-12.19755800	5.50325500	-0.66197800
H	-11.14952800	3.48793900	2.36028100
H	-12.74654500	2.84501000	1.99004900
H	-11.30154000	1.90401000	1.57987100
H	-6.20918700	-4.06266300	-1.94785500
H	-6.15981900	-5.41518500	-0.80384600
H	-7.67390300	-4.55592800	-1.10530300
H	-10.19664100	-0.59675600	-2.81750800
H	-8.59821500	-1.19594200	-3.27157700
H	-9.18638200	0.35000400	-3.90638700
C	2.17330200	-2.51388900	5.44601200
H	2.99519200	-2.18297200	6.09260200
H	1.28211000	-2.63642300	6.07361900
H	2.43731500	-3.49442300	5.03865600
C	0.95442200	2.30899700	-4.69018300
H	1.83968700	2.43225800	-5.32598300
H	0.12581300	1.98147800	-5.32989400
H	0.69590500	3.28858000	-4.27705300
H	10.34855300	-0.20970400	-2.80126700
H	8.92801100	5.21959600	-1.69391300

Heme a (Ox. Deprotonated)

C	1.18904900	1.35759900	-3.49480000
N	1.55715900	0.04655600	-3.71309200
C	1.15685000	1.49360800	-2.14373100
C	1.73203500	-0.55570500	-2.53046200
N	1.49756200	0.29399700	-1.55178400
C	2.04616700	-1.55288300	4.24373100
N	1.72233400	-0.22971000	4.45839800
C	2.05480000	-1.69818500	2.89331100
C	1.54998700	0.37050000	3.27425100
N	1.74439400	-0.49202000	2.29797800
Fe	1.60075800	-0.10376400	0.37289200
C	4.86055900	0.79672200	0.35097300
C	2.45874600	-3.34587000	-0.32337400
C	-1.65894300	-0.97723200	0.37270500
C	0.72169200	3.12876300	1.08403900
N	3.32174800	-1.09597000	0.07186400
C	4.57825500	-0.54402900	0.10862600
C	5.57564800	-1.53046900	-0.13861300
C	4.88774000	-2.71029500	-0.33646200

C	3.49498800	-2.40957600	-0.19690500
C	5.49229900	-4.00169600	-0.63247800
O	4.89462200	-5.04775900	-0.82557800
C	7.05138500	-1.28989900	-0.21603600
C	7.51213900	-0.87028500	-1.61200900
C	9.03850800	-0.67256000	-1.73684600
O	9.43746600	-0.24677900	-2.84697700
O	9.73718300	-0.95897200	-0.73594400
N	0.61053900	-1.80651100	0.08764700
C	1.13667700	-3.05196000	-0.19083100
C	0.07422600	-4.03215000	-0.33339400
C	-1.08531400	-3.36078000	-0.15594700
C	-0.72875100	-1.97428800	0.11418400
C	0.31753700	-5.48202400	-0.60636600
N	-0.10080000	0.87427800	0.67739800
C	-1.34865600	0.33485000	0.63894800
C	-2.35952400	1.34650300	0.92100400
C	-1.69243900	2.50689200	1.11738900
C	-0.27478800	2.20301500	0.96841300
C	-3.82637600	1.07961800	0.99979000
C	-2.21526100	3.83721100	1.44796000
C	-3.30915100	4.37707500	0.91889200
N	2.58343200	1.61704200	0.65291900
C	2.08108300	2.83824300	0.92750400
C	3.12535900	3.82456500	1.04657300
C	4.29795700	3.16981100	0.83880100
C	3.94940000	1.78755300	0.59385100
C	2.89776800	5.27447300	1.33530400
C	5.68288100	3.73696200	0.80386900
C	6.14032600	4.10334400	-0.60733500
C	7.52188800	4.78819500	-0.67352000
O	8.06269800	5.10014000	0.41418800
O	7.97160300	4.98275700	-1.82845600
C	-2.49636300	-3.87711700	-0.16586300
O	-2.59692700	-4.90631600	-1.14135200
C	-2.90852600	-4.37494000	1.22198400
C	-4.37915700	-4.79918100	1.31319900
C	-5.31752900	-3.64397100	1.10185200
C	-6.22362600	-3.46437900	0.13926200
C	-7.04566900	-2.19734500	0.09715600
C	-6.78466800	-1.33350400	-1.14749200
C	-7.51889700	-0.02496000	-1.07585900
C	-8.50933800	0.42189800	-1.84952200
C	-9.12948800	1.77404100	-1.57952400

C	-10.58992700	1.70342500	-1.10505300
C	-11.16645100	3.07100900	-0.87377900
C	-11.61294600	3.60657800	0.26255700
C	-12.15106600	5.01354200	0.29260700
C	-11.64016200	2.91088200	1.59694300
C	-6.51131000	-4.45200000	-0.96202500
C	-9.09264600	-0.33068000	-3.01670200
H	1.67789100	-0.39879900	-4.61676500
H	0.91209100	2.36826500	-1.55733400
H	2.02119100	-1.59184300	-2.41672200
H	1.63081600	0.22522800	5.36071600
H	2.26148700	-2.58448500	2.30989700
H	1.28808000	1.41356500	3.15780600
H	5.90837300	1.08289200	0.34550800
H	2.75435600	-4.36549600	-0.54498700
H	-2.70911200	-1.24654300	0.36941000
H	0.44855600	4.15701200	1.29948600
H	6.59916600	-3.99532900	-0.68014100
H	7.34960500	-0.52216400	0.50654200
H	7.60380000	-2.18773300	0.07744000
H	7.01664200	0.06122400	-1.91830800
H	7.20584600	-1.62440900	-2.35116300
H	-0.62695500	-6.02255100	-0.67826600
H	0.86327300	-5.61932800	-1.54834200
H	0.92465400	-5.93152700	0.18920700
H	-4.33815500	1.89522300	1.51946800
H	-4.27394200	0.98928000	0.00132600
H	-4.03499300	0.14827600	1.53871200
H	-1.64818300	4.41139300	2.18229900
H	-3.89579400	3.86693400	0.15673700
H	-3.64875600	5.36594600	1.22075800
H	2.33121000	5.41815500	2.26389900
H	2.33397400	5.76237000	0.52991000
H	3.84805900	5.80636900	1.44098300
H	5.73618600	4.63512000	1.42713400
H	6.39916400	3.03381100	1.24601300
H	5.40772800	4.78034700	-1.07054200
H	6.16564600	3.21338900	-1.25093600
H	-3.17004200	-3.05823300	-0.45572000
H	-3.53729600	-5.09523200	-1.28366200
H	-2.71475300	-3.57609900	1.95141800
H	-2.25783100	-5.21755200	1.49055500
H	-4.57089400	-5.61742800	0.60937200
H	-4.54846900	-5.22072600	2.31401800

H	-5.20900800	-2.84448800	1.84075500
H	-8.11597600	-2.45221500	0.12359700
H	-6.84229200	-1.59853800	0.99492200
H	-7.05293800	-1.89124800	-2.05066200
H	-5.70341900	-1.13765300	-1.21047700
H	-7.19378500	0.62486300	-0.25794500
H	-9.08770500	2.38371000	-2.49557900
H	-8.54018700	2.30562300	-0.82133800
H	-11.19434800	1.19117500	-1.86728100
H	-10.64096200	1.08472500	-0.20199700
H	-11.20263700	3.69836900	-1.76915300
H	-11.57699800	5.63770600	0.99157700
H	-13.19266000	5.02741900	0.64284000
H	-12.11663300	5.48600200	-0.69524600
H	-11.07108000	3.48745000	2.33905300
H	-12.67006800	2.84630700	1.97427900
H	-11.22785100	1.89895000	1.56892400
H	-6.13262100	-4.09164400	-1.92807300
H	-6.07726000	-5.43916300	-0.77832200
H	-7.59444800	-4.58604400	-1.08130500
H	-10.13005500	-0.62897000	-2.81665300
H	-8.53325500	-1.23603900	-3.26600700
H	-9.11561300	0.30837400	-3.90976000
C	2.30794500	-2.51456700	5.35020800
H	3.14750200	-2.18520900	5.97364100
H	1.43007900	-2.62695800	5.99732100
H	2.55492100	-3.49677900	4.93740400
C	0.91407400	2.31824500	-4.59907700
H	1.79730200	2.46077400	-5.23279100
H	0.09354400	1.96827500	-5.23644800
H	0.63241300	3.29003800	-4.18383000

Heme a (Red, Deprotonated)

C	1.24482700	1.32458100	-3.61570500
N	1.59282900	0.00821800	-3.81641400
C	1.20534500	1.47258200	-2.26235300
C	1.74704700	-0.57680500	-2.60995000
N	1.52003400	0.28186200	-1.64553300
C	1.96243100	-1.50007400	4.32813600
N	1.62275600	-0.18061300	4.52242100
C	2.00597500	-1.65344900	2.97551200
C	1.47754500	0.40104100	3.31315900
N	1.70263800	-0.46284600	2.35289000
Fe	1.61125800	-0.09108700	0.35343800

C	4.88805600	0.79263200	0.37386300
C	2.44689700	-3.33100000	-0.29467500
C	-1.67431400	-0.94637800	0.32954900
C	0.74501500	3.14116500	1.00915400
N	3.34479200	-1.09425900	0.08997300
C	4.60382200	-0.55835400	0.13682400
C	5.59697100	-1.55646400	-0.08983300
C	4.89896600	-2.73282000	-0.28739900
C	3.50007400	-2.40860000	-0.16609400
C	5.49705600	-4.02305700	-0.56274600
O	4.90545500	-5.07634200	-0.75785100
C	7.07727900	-1.33034900	-0.15209600
C	7.56085900	-0.92115900	-1.54316100
C	9.08882200	-0.73605500	-1.65916400
O	9.49857700	-0.30966300	-2.76598000
O	9.78256300	-1.03222600	-0.65720600
N	0.58750600	-1.79926700	0.07676900
C	1.11677400	-3.04119000	-0.18205600
C	0.05354200	-4.03002300	-0.32666700
C	-1.10722800	-3.35705400	-0.16609400
C	-0.74665000	-1.96188000	0.09278200
C	0.29505500	-5.48443000	-0.58344700
N	-0.11592400	0.90183300	0.62056100
C	-1.36443100	0.37385200	0.57740400
C	-2.37416100	1.40463500	0.83095600
C	-1.69542100	2.55943500	1.01591000
C	-0.27200000	2.22802400	0.88875300
C	-3.84585200	1.15661000	0.89778400
C	-2.20512400	3.90011500	1.32051700
C	-3.30557200	4.44252400	0.80547500
N	2.61670600	1.62962000	0.62728100
C	2.11006200	2.84636000	0.88212300
C	3.16222000	3.84216500	1.01555100
C	4.33455400	3.18751200	0.83525500
C	3.98343500	1.79514500	0.59254600
C	2.92946600	5.29457700	1.29005000
C	5.72376000	3.74870800	0.82346400
C	6.22670500	4.07737000	-0.58123900
C	7.62172400	4.73403800	-0.63191000
O	8.15886400	5.03529500	0.46101200
O	8.08996500	4.91872800	-1.78187800
C	-2.51793500	-3.87254300	-0.17919800
O	-2.62824100	-4.89738300	-1.16196200
C	-2.93368700	-4.37909600	1.20434900

C	-4.40339900	-4.80738700	1.29495800
C	-5.34548200	-3.65372800	1.09249900
C	-6.25306500	-3.46924300	0.13235000
C	-7.07798100	-2.20374800	0.10027100
C	-6.81517500	-1.32662400	-1.13472300
C	-7.55377100	-0.02118800	-1.05229700
C	-8.54681700	0.42807200	-1.82122200
C	-9.17237200	1.77527000	-1.53924800
C	-10.63108400	1.69394600	-1.06113900
C	-11.21363100	3.05680500	-0.81743600
C	-11.65922900	3.58127900	0.32441900
C	-12.20422100	4.98527100	0.36713900
C	-11.67882500	2.87508200	1.65340900
C	-6.53864400	-4.44900500	-0.97654800
C	-9.12846600	-0.31741700	-2.99377700
H	1.71414600	-0.44969400	-4.71219600
H	0.97057400	2.35775700	-1.68515900
H	2.01973700	-1.61590600	-2.47630000
H	1.50264500	0.28183100	5.41602300
H	2.23547700	-2.54289300	2.40288100
H	1.21053700	1.44100700	3.17437800
H	5.93933700	1.07123400	0.38194600
H	2.73819400	-4.35565000	-0.50371400
H	-2.72710200	-1.21157400	0.32096000
H	0.47872000	4.17567300	1.20890900
H	6.60603500	-4.02107000	-0.59390500
H	7.37300800	-0.56000000	0.56956600
H	7.62067600	-2.23050400	0.15227700
H	7.07530500	0.01233300	-1.85954200
H	7.25480700	-1.67638700	-2.28173100
H	-0.64988400	-6.02297500	-0.67123100
H	0.86094500	-5.63456700	-1.51203000
H	0.88383100	-5.93356400	0.22674400
H	-4.35373100	1.97355800	1.42039500
H	-4.29078600	1.07887500	-0.10346800
H	-4.06991200	0.22240800	1.42609700
H	-1.61800900	4.48881900	2.02790600
H	-3.91513100	3.92418900	0.06717900
H	-3.62821600	5.44103800	1.09477500
H	2.34623600	5.44564500	2.20778800
H	2.37590800	5.77765000	0.47391100
H	3.87655500	5.83097700	1.40718400
H	5.76706400	4.66143800	1.42706000
H	6.42421400	3.05195600	1.30073500

H	5.51855700	4.75597500	-1.07909500
H	6.25077900	3.17258500	-1.20425700
H	-3.19103800	-3.05042200	-0.46218200
H	-3.57025800	-5.08821600	-1.28973500
H	-2.74149700	-3.58231700	1.93648300
H	-2.28163500	-5.22153600	1.47063400
H	-4.59455700	-5.62152300	0.58601300
H	-4.57160200	-5.23592800	2.29316600
H	-5.23812200	-2.85897800	1.83667600
H	-8.14790300	-2.46091700	0.12093300
H	-6.87857900	-1.61338800	1.00451900
H	-7.07843100	-1.87609900	-2.04445900
H	-5.73441400	-1.12662200	-1.19159000
H	-7.23027600	0.62315400	-0.22943100
H	-9.13609800	2.39221300	-2.45065400
H	-8.58343500	2.30373200	-0.77860300
H	-11.23523800	1.18471200	-1.82560700
H	-10.67642000	1.06791500	-0.16283500
H	-11.25566300	3.69097000	-1.70775000
H	-11.63106700	5.60680900	1.06919500
H	-13.24477400	4.99122000	0.72069100
H	-12.17521200	5.46560000	-0.61708900
H	-11.11020400	3.44862700	2.39821000
H	-12.70720200	2.80249800	2.03346300
H	-11.26163200	1.86545600	1.61618300
H	-6.15984700	-4.08098500	-1.93969000
H	-6.10273700	-5.43666200	-0.79986700
H	-7.62148200	-4.58422400	-1.09757400
H	-10.16463900	-0.62101200	-2.79524500
H	-8.56601400	-1.21880400	-3.25050700
H	-9.15446600	0.32847200	-3.88183200
C	2.20404900	-2.45179200	5.44921600
H	3.02769100	-2.11664800	6.09142300
H	1.31370200	-2.56683100	6.07949000
H	2.46549800	-3.43660300	5.05056100
C	0.99302900	2.27902100	-4.73225300
H	1.88101400	2.40362300	-5.36401300
H	0.17054400	1.94014900	-5.37399600
H	0.72500000	3.26013300	-4.32887400

Heme b (Ox, Protonated)

Fe	0.91442500	-0.02670700	0.13690700
C	4.26440000	-0.11155000	-0.36623000
H	5.33528200	-0.15250600	-0.54318900

N	2.29289900	-1.48083000	0.04743700
C	3.60512200	-1.33690100	-0.16054700
C	2.08239600	-2.83253900	0.19321200
C	4.29486000	-2.63220900	-0.16369700
C	3.34548600	-3.56554500	0.06666700
C	0.87085300	-3.42066600	0.42431300
H	0.84141100	-4.50251300	0.51701200
N	-0.49853800	-1.40847600	0.45519900
C	-0.34883000	-2.73218700	0.54524500
C	-1.84370100	-1.16975100	0.63823400
C	-1.63036400	-3.40746400	0.79123300
C	-2.55937600	-2.43668500	0.84948000
C	-2.43513800	0.05599000	0.63459600
H	-3.50681800	0.09787400	0.80596500
N	-0.46456300	1.42404800	0.22739500
C	-1.77207800	1.28487900	0.43132400
C	-0.25070600	2.78229200	0.07933200
C	-2.46536500	2.58240100	0.42368700
C	-1.51856000	3.51208900	0.20229200
C	0.95386400	3.36863300	-0.15015100
H	0.97789400	4.44922200	-0.25811600
N	2.32848200	1.35886500	-0.18242900
C	2.18244300	2.67980900	-0.26878700
C	3.67388500	1.11714800	-0.37112300
C	3.46117100	3.35163600	-0.52759500
C	4.39383300	2.37570300	-0.58346700
N	0.61460500	-0.18835900	-1.86859700
C	0.46633200	0.79775700	-2.72211000
C	0.52186200	-1.34706200	-2.60956900
N	0.28378600	0.32468600	-3.97059600
C	0.31578400	-1.04948100	-3.92200500
H	0.48299400	1.85282800	-2.48185100
H	0.60992800	-2.32235300	-2.14938400
H	0.14512400	0.89104800	-4.79963400
C	-4.03773700	-2.56167900	1.04633400
H	-4.26452600	-3.49454100	1.57345200
H	-4.40770000	-1.75586200	1.69130600
C	-3.94054700	2.76822600	0.59969600
H	-4.14615600	3.78536900	0.94975700
H	-4.32076900	2.10141600	1.38274300
C	-4.71421000	2.52192700	-0.69394100
H	-4.39027600	3.21380400	-1.48165000
H	-4.53235600	1.51355600	-1.08797000
C	-4.79938500	-2.53979700	-0.27745300

H	-4.48359100	-3.36757100	-0.92504500
H	-4.59604000	-1.62177300	-0.84345300
C	-6.28697000	-2.64350100	-0.08312100
C	-6.19828900	2.68473500	-0.51289100
O	-6.85322600	-2.73462200	0.98201700
O	-6.86678600	2.48686400	-1.65642000
O	-6.75486600	2.96042100	0.52523800
O	-6.94649800	-2.62433400	-1.24865600
C	-1.78921300	-4.88540200	0.93148100
H	-2.82840600	-5.15500100	1.14175200
H	-1.48944600	-5.40626600	0.01336000
H	-1.16910600	-5.27775000	1.74714800
C	-1.64773900	4.99429500	0.08617800
H	-1.34127600	5.34063700	-0.90877000
H	-2.67979800	5.31853800	0.24816500
H	-1.01414700	5.50698300	0.82033800
C	3.48432900	-5.04361900	0.21197100
H	2.89127000	-5.41774800	1.05435900
H	3.14252500	-5.56930200	-0.68908200
H	4.52989900	-5.31845500	0.38040400
C	5.86860500	2.48604000	-0.77881300
H	6.41163600	1.82913300	-0.08979900
H	6.16027900	2.20495500	-1.79908100
H	6.20441700	3.51333500	-0.60847100
C	5.73957500	-2.80150300	-0.35408500
C	6.30282500	-3.77012800	-1.07019500
H	6.38340300	-2.06965100	0.13680300
H	7.38489300	-3.84876500	-1.15510500
H	5.71164000	-4.50798000	-1.61007600
C	3.62135700	4.80380200	-0.65909100
C	4.41469500	5.40373400	-1.54176200
H	3.03877600	5.42079200	0.02728300
H	4.50088100	6.48809700	-1.57292000
H	4.99271100	4.84267600	-2.27420900
C	0.14777200	-1.91037800	-5.12644800
H	-0.82377600	-1.74294800	-5.60691400
H	0.92869400	-1.71520000	-5.87129300
H	0.20761200	-2.96483500	-4.84116400
N	1.21549600	0.13378900	2.14220800
C	1.36785300	-0.85507600	2.99182000
C	1.30323300	1.28992700	2.88777400
H	1.35483500	-1.90916800	2.74704900
C	1.51044800	0.98809200	4.19905900
H	1.21073500	2.26688000	2.43197400

H	1.68889300	-0.95509400	5.06907600
N	1.54807300	-0.38609500	4.24220000
C	1.67493800	1.84501900	5.40678500
H	0.89458200	1.64412500	6.15069600
H	2.64699500	1.67936000	5.88682900
H	1.61126800	2.90032500	5.12549100
H	-7.81785800	2.60752500	-1.46704900
H	-7.90053800	-2.69491600	-1.04926300

Heme b (Red, Protonated)

Fe	0.91404300	-0.02877600	0.13068200
C	4.26459600	-0.10781500	-0.37832700
H	5.33655600	-0.13347800	-0.55752400
N	2.29954300	-1.48790000	0.03043000
C	3.63110100	-1.32583500	-0.18300100
C	2.10213900	-2.82473900	0.16731300
C	4.30788800	-2.61118600	-0.19378300
C	3.34759200	-3.55082800	0.03559200
C	0.86909400	-3.42570400	0.40392800
H	0.85245400	-4.50963100	0.48921600
N	-0.50543700	-1.41517200	0.45030200
C	-0.33511600	-2.76632800	0.53207700
C	-1.82743500	-1.19389400	0.63817000
C	-1.60643300	-3.42988700	0.77816000
C	-2.53791000	-2.45000400	0.84622500
C	-2.43738900	0.05321600	0.64305300
H	-3.51002200	0.07916100	0.81888800
N	-0.46998100	1.42467400	0.23192800
C	-1.79974300	1.26561400	0.44854100
C	-0.26924500	2.76390300	0.09300100
C	-2.47954500	2.55733600	0.45064500
C	-1.52596500	3.48958600	0.22547000
C	0.94852300	3.36810100	-0.14365700
H	0.95441700	4.45037600	-0.24553600
N	2.33062100	1.36511200	-0.18921600
C	2.16579400	2.70487200	-0.27053300
C	3.66245200	1.13673200	-0.37902000
C	3.43514800	3.36683500	-0.53006600
C	4.37384000	2.38250500	-0.58950700
N	0.60689400	-0.17883900	-1.87586900
C	0.49777800	0.81629900	-2.72245300
C	0.46687100	-1.32915200	-2.62054600
N	0.29426600	0.35722900	-3.97541000
C	0.27109000	-1.01813600	-3.93205700

H	0.55871300	1.86712200	-2.46910800
H	0.51681500	-2.30602100	-2.15693100
H	0.17750900	0.93162300	-4.80185600
C	-4.01682500	-2.58189500	1.05091900
H	-4.24205400	-3.52102900	1.56875800
H	-4.38748200	-1.78417100	1.70610500
C	-3.95423400	2.75552800	0.62963600
H	-4.15503000	3.76856000	0.99608800
H	-4.34254200	2.07737300	1.39907700
C	-4.73009200	2.53591600	-0.66714300
H	-4.39488700	3.23174800	-1.44694400
H	-4.55520900	1.52949800	-1.06910600
C	-4.78955600	-2.54617900	-0.26573200
H	-4.47239300	-3.36223100	-0.92778900
H	-4.59118000	-1.61880500	-0.81843400
C	-6.27594700	-2.65926000	-0.07136900
C	-6.21302300	2.71260800	-0.49480400
O	-6.84341700	-2.76931100	0.99176100
O	-6.87721700	2.53551100	-1.64555500
O	-6.77719800	2.98192300	0.54133100
O	-6.93912100	-2.62577900	-1.23582600
C	-1.78384500	-4.90893900	0.91269900
H	-2.82104200	-5.16732300	1.14887000
H	-1.51690700	-5.43117600	-0.01515300
H	-1.14927600	-5.31965500	1.70824200
C	-1.66615200	4.97453200	0.11615200
H	-1.38677900	5.33093200	-0.88379200
H	-2.69624600	5.29409500	0.30347600
H	-1.02027500	5.49348500	0.83571600
C	3.50065800	-5.03193700	0.17494300
H	2.84805200	-5.43088800	0.96004800
H	3.25091700	-5.56042800	-0.75511100
H	4.53315900	-5.29246300	0.43031200
C	5.85056200	2.50872300	-0.78714800
H	6.40165900	1.79723400	-0.16159200
H	6.14230000	2.31998000	-1.82915100
H	6.19080700	3.51753900	-0.53109100
C	5.75039400	-2.79017600	-0.38532300
C	6.33100100	-3.80210000	-1.02649300
H	6.39213500	-2.01612100	0.04020500
H	7.41454700	-3.86709600	-1.10805100
H	5.75517100	-4.59116400	-1.50694100
C	3.60335800	4.81746300	-0.65907900
C	4.45452500	5.43268800	-1.47712100

H	2.96596700	5.43393900	-0.02202300
H	4.52849700	6.51853500	-1.49714000
H	5.09479500	4.88500600	-2.16645100
C	0.06689600	-1.86606600	-5.14055800
H	-0.89858500	-1.66009100	-5.61873100
H	0.85264600	-1.69877800	-5.88741600
H	0.08654400	-2.92356500	-4.86042900
N	1.22216800	0.12165100	2.13692000
C	1.32960500	-0.87251900	2.98482500
C	1.36074500	1.27284400	2.88053200
H	1.26799600	-1.92358700	2.73273100
C	1.55414700	0.96326800	4.19273600
H	1.31070800	2.24920900	2.41581200
H	1.64622000	-0.98550100	5.06492100
N	1.53089800	-0.41205500	4.23762100
C	1.75622800	1.81253400	5.40065600
H	0.96951000	1.64563100	6.14657800
H	2.72114200	1.60751000	5.88038200
H	1.73649100	2.86973100	5.11940600
H	-7.89188300	-2.70389500	-1.03346600
H	-7.82766900	2.66419700	-1.45876100

Heme b (Ox. Deprotonated)

Fe	0.86840100	-0.02853500	0.14368600
C	4.20654600	-0.08917600	-0.37897600
H	5.27591000	-0.11092100	-0.56576000
N	2.24471800	-1.47813200	0.04951900
C	3.57751800	-1.30208100	-0.17490500
C	2.06676600	-2.82303000	0.18885700
C	4.26125300	-2.57491700	-0.18654500
C	3.31292300	-3.52750800	0.05139400
C	0.84669900	-3.44097800	0.42925800
H	0.83636000	-4.52333300	0.51715800
N	-0.51861600	-1.42047500	0.46021700
C	-0.35148100	-2.77918200	0.54910900
C	-1.84798300	-1.20441800	0.63310300
C	-1.61957900	-3.43423700	0.78407900
C	-2.55632200	-2.45664400	0.83684500
C	-2.46858200	0.03142500	0.62807200
H	-3.54256700	0.05024500	0.78286700
N	-0.49761900	1.41211900	0.23651900
C	-1.83381700	1.24020800	0.44073800
C	-0.31564100	2.76230200	0.10305200
C	-2.52547400	2.51825600	0.43966700

C	-1.57705800	3.46213000	0.22801900
C	0.88732100	3.38475300	-0.12863000
H	0.88669400	4.46588500	-0.22828600
N	2.26133200	1.37478900	-0.17740500
C	2.09997600	2.71987000	-0.25669600
C	3.59607500	1.14712300	-0.37566900
C	3.36060100	3.37443800	-0.51922400
C	4.29976800	2.38881900	-0.58624700
N	0.58586300	-0.18908100	-1.79827300
C	0.45894900	0.81266400	-2.64345200
C	0.46863000	-1.34799200	-2.53850400
N	0.26725300	0.34529600	-3.88360100
C	0.26909300	-1.03320700	-3.84474300
H	0.49829300	1.86404000	-2.39155300
H	0.53504400	-2.32463200	-2.07958200
H	0.13898100	0.91908400	-4.71023800
C	-4.03798900	-2.58908900	1.00856100
H	-4.26703200	-3.51106400	1.55467000
H	-4.43057800	-1.77788300	1.63189600
C	-4.00460300	2.69914200	0.58812400
H	-4.22058100	3.69878500	0.97981200
H	-4.40501900	2.00236200	1.33400000
C	-4.76042800	2.51689500	-0.72713300
H	-4.36194900	3.20715700	-1.48441300
H	-4.59097500	1.50928900	-1.13220100
C	-4.79038800	-2.60135800	-0.32116100
H	-4.43649300	-3.43187700	-0.94753200
H	-4.56668000	-1.68824100	-0.89188700
C	-6.32405000	-2.71530300	-0.19276200
C	-6.28386200	2.74476100	-0.63191700
O	-6.80969900	-2.68828800	0.96324100
O	-6.92203200	2.56710400	-1.69767100
O	-6.74734300	3.08767700	0.48180200
O	-6.94851900	-2.81879700	-1.27621000
C	-1.79643700	-4.91242800	0.92136800
H	-2.83685700	-5.16437200	1.14721400
H	-1.52153700	-5.43643700	-0.00282500
H	-1.17148400	-5.31990700	1.72581500
C	-1.73533100	4.94488000	0.12191500
H	-1.46258000	5.30628800	-0.87780600
H	-2.77054000	5.24427400	0.31071100
H	-1.09788200	5.47049800	0.84377200
C	3.48414700	-5.00606200	0.18909300
H	2.85151400	-5.41138700	0.98660300

H	3.22359800	-5.53448500	-0.73736600
H	4.52407200	-5.25212200	0.42587300
C	5.77386600	2.51677400	-0.79470700
H	6.33191900	1.81968500	-0.15990400
H	6.05575000	2.31117600	-1.83569300
H	6.10852500	3.53157000	-0.55797500
C	5.70449900	-2.74064300	-0.38945200
C	6.27403100	-3.73449800	-1.06594300
H	6.34742600	-1.97942400	0.05539100
H	7.35617500	-3.79912100	-1.16200000
H	5.69005100	-4.50758700	-1.56222400
C	3.52813900	4.82626600	-0.64828000
C	4.35159400	5.43091200	-1.50017200
H	2.92027800	5.44409500	0.01490300
H	4.43333500	6.51584200	-1.52650500
H	4.95997700	4.87529300	-2.21168500
C	0.08071200	-1.87843700	-5.05633900
H	-0.88796200	-1.68348400	-5.53152500
H	0.86519100	-1.69125500	-5.79904800
H	0.11807400	-2.93619900	-4.78068700
N	1.17402700	0.13240700	2.08350000
C	1.30100800	-0.86718700	2.93114200
C	1.29196500	1.29303600	2.82103500
H	1.25794200	-1.91916300	2.68263300
C	1.49262400	0.98135300	4.12786900
H	1.22285100	2.26855900	2.36016100
H	1.62168300	-0.96889900	4.99808400
N	1.49427700	-0.39700700	4.17002100
C	1.68152500	1.82943200	5.33742100
H	0.89782900	1.64325800	6.08120500
H	2.65075600	1.63639300	5.81228800
H	1.64291300	2.88655200	5.05947900

Heme b (Red, Deprotonated)

Fe	0.87805700	-0.02778800	0.13460900
C	4.22811100	-0.09967900	-0.38743700
H	5.29935000	-0.12331000	-0.57213700
N	2.26845900	-1.48434500	0.03725100
C	3.60019600	-1.31902700	-0.18389300
C	2.07555100	-2.81936500	0.18216900
C	4.27877100	-2.60165500	-0.19154000
C	3.32059400	-3.54278800	0.04832400
C	0.84157700	-3.42314000	0.42611600
H	0.82837800	-4.50674600	0.51714200

N	-0.53718100	-1.41646500	0.45874300
C	-0.36280000	-2.76817000	0.54923700
C	-1.86117100	-1.20049600	0.63521100
C	-1.63581300	-3.43300800	0.79228000
C	-2.57343200	-2.45881500	0.84620200
C	-2.47591900	0.04427300	0.62953400
H	-3.55089300	0.06628900	0.78793100
N	-0.51032600	1.42086000	0.23146500
C	-1.84078900	1.25879800	0.43792600
C	-0.31356500	2.76235700	0.09260400
C	-2.52988000	2.54890500	0.43556300
C	-1.57554400	3.48222500	0.21931000
C	0.90069900	3.36854800	-0.14410300
H	0.90344700	4.45080900	-0.24739700
N	2.29111700	1.37015900	-0.19244500
C	2.12238600	2.70789000	-0.27498100
C	3.62414300	1.14424500	-0.38901000
C	3.38713300	3.37271800	-0.54221800
C	4.32946800	2.39008000	-0.60572300
N	0.56737600	-0.18496700	-1.87107300
C	0.44362400	0.80754700	-2.71846700
C	0.43140200	-1.33779000	-2.61233500
N	0.23455700	0.34441600	-3.96924300
C	0.22299800	-1.03108200	-3.92296900
H	0.49667300	1.85921200	-2.46664000
H	0.49199600	-2.31295900	-2.14628200
H	0.10554500	0.91617000	-4.79564900
C	-4.05551900	-2.59305300	1.02464600
H	-4.28470200	-3.51848300	1.56621300
H	-4.44431500	-1.78438600	1.65458200
C	-4.00809200	2.73937900	0.59254400
H	-4.22044900	3.74947900	0.95991900
H	-4.40120900	2.06154300	1.36056000
C	-4.78279300	2.52287300	-0.70635100
H	-4.39706800	3.19752900	-1.48473000
H	-4.61216400	1.50762700	-1.09075300
C	-4.81862100	-2.59521000	-0.29884100
H	-4.46745500	-3.42005100	-0.93456200
H	-4.59600100	-1.67754900	-0.86288900
C	-6.35154700	-2.70952800	-0.16953400
C	-6.30593400	2.74463800	-0.60178200
O	-6.83926500	-2.68193100	0.98597500
O	-6.96071700	2.49383100	-1.64331200
O	-6.75756600	3.15808900	0.49316500

O	-6.97805300	-2.81365900	-1.25262600
C	-1.80946400	-4.91208700	0.93440100
H	-2.84707100	-5.16816100	1.17138400
H	-1.54182400	-5.44039200	0.00984100
H	-1.17497600	-5.31891700	1.73241200
C	-1.72059900	4.96703700	0.11046600
H	-1.45080200	5.32621300	-0.89145500
H	-2.75126400	5.27971200	0.30608300
H	-1.07179100	5.48988100	0.82497100
C	3.47859100	-5.02297800	0.19464100
H	2.82575400	-5.42085900	0.98012900
H	3.23298300	-5.55740400	-0.73325400
H	4.51145200	-5.27901600	0.45350600
C	5.80450500	2.52248700	-0.81336600
H	6.36271300	1.80413900	-0.20216800
H	6.08889500	2.34916000	-1.86016000
H	6.14459500	3.52866900	-0.54645300
C	5.72024800	-2.77949800	-0.39057700
C	6.30068800	-3.79726900	-1.02292000
H	6.36267900	-1.99829700	0.02075600
H	7.38395700	-3.85928900	-1.11060400
H	5.72486900	-4.59445200	-1.48974900
C	3.55085600	4.82335200	-0.67440400
C	4.40263000	5.44175800	-1.48976400
H	2.90848300	5.43876600	-0.04123400
H	4.47166700	6.52794400	-1.51053600
H	5.04823500	4.89655900	-2.17604200
C	0.01545500	-1.88300100	-5.12811900
H	-0.95563700	-1.68564300	-5.59855300
H	0.79349400	-1.71121200	-5.88203500
H	0.04561800	-2.93975200	-4.84606100
N	1.18870100	0.12979700	2.14078200
C	1.29165600	-0.86155200	2.99231600
C	1.33458100	1.28306000	2.87956200
H	1.22326400	-1.91309800	2.74382700
C	1.52813300	0.97771100	4.19278700
H	1.28858400	2.25754200	2.41041600
H	1.61015800	-0.96807300	5.07271700
N	1.49742800	-0.39733700	4.24325300
C	1.73671800	1.83059000	5.39707800
H	0.95070400	1.67050100	6.14526300
H	2.70158500	1.62283700	5.87580500
H	1.72155200	2.88679400	5.11178800

Heme c (Ox, Protonated)

Fe	-0.55237200	-0.03206400	0.21113300
C	-4.13549400	-2.30044500	-0.20225500
C	-3.24762100	-3.32874700	-0.09803300
C	-1.95176700	-2.72305500	0.05885800
C	-3.36256000	-1.08026400	-0.12067000
N	-2.03863100	-1.36450600	0.04177300
C	-3.89439100	0.19272200	-0.19822100
C	-3.18853600	1.37428800	-0.12433400
N	-1.83893100	1.49803600	0.03993800
C	-1.58697900	2.83386400	0.06237800
C	-3.81216100	2.67340600	-0.21021000
C	-2.81323000	3.58785100	-0.08792400
C	-0.31903400	3.38604100	0.19987600
C	0.83649000	2.65580200	0.34383100
N	0.92128000	1.29084600	0.37633400
C	3.02366100	2.22813300	0.61547500
C	2.14796200	3.25300400	0.48818000
C	2.07679000	-1.43829500	0.55503900
N	0.73438700	-1.54206200	0.38521300
C	0.46684800	-2.88629000	0.36612100
C	1.68370000	-3.65226900	0.52718000
C	2.68795100	-2.75185700	0.64702800
C	-0.77773600	-3.44644200	0.21314000
C	2.78873100	-0.25532800	0.63368000
C	2.24309800	1.00676100	0.54237000
H	-4.96722700	0.28399000	-0.33046300
H	-0.22833000	4.46596600	0.17827700
H	-0.84740900	-4.53004000	0.21532200
H	3.86173800	-0.32668700	0.78091000
C	4.51317800	2.29109100	0.76267700
H	4.86473400	1.52039300	1.45843500
H	4.80131800	3.24824500	1.21045900
C	5.23402000	2.13134100	-0.57417000
H	4.93035400	2.91505400	-1.27986600
H	4.97847800	1.17878000	-1.05607100
C	4.15518300	-3.01049000	0.80373500
H	4.60164300	-2.28848600	1.49742100
H	4.31176400	-3.99446100	1.25860000
C	4.89804300	-2.95608600	-0.52957500
H	4.77310100	-1.98165800	-1.01905500
H	4.49729900	-3.69788500	-1.23219500
C	1.74874300	-5.14533100	0.54127500
H	1.10626800	-5.56682100	1.32401600

H	1.41841900	-5.56728000	-0.41606900
H	2.76884800	-5.49641600	0.72275100
C	2.41208400	4.72394500	0.48420200
H	2.11737100	5.17892700	-0.46967500
H	1.85053600	5.23364600	1.27684800
H	3.47394800	4.93726200	0.63835600
C	-2.96962700	5.08341600	-0.06604800
H	-4.02322900	5.29866500	-0.25948600
C	-5.28144900	2.88570700	-0.38948600
H	-5.64350000	2.39784600	-1.30252900
H	-5.84840700	2.46931600	0.45223100
H	-5.53488900	3.94673100	-0.46266300
C	-5.63116400	-2.36401500	-0.35307000
H	-6.02649500	-1.34667100	-0.38315700
C	-3.48387300	-4.80720900	-0.12306500
H	-2.75162000	-5.31481600	-0.76133200
H	-3.40014400	-5.24118300	0.88177000
H	-4.47592500	-5.04648600	-0.51275700
C	-6.32106900	-3.09467300	0.79693500
H	-6.07402900	-2.60242400	1.74541200
H	-7.40968100	-3.06673900	0.67353500
H	-6.01115500	-4.14169100	0.86183400
C	-2.60502200	5.71841800	1.27538400
H	-2.81599400	6.79366400	1.26111400
H	-3.20799100	5.26430600	2.07107500
H	-1.54893400	5.58196200	1.52653300
S	-6.01636400	-3.09949000	-1.98986300
H	-7.34949500	-2.94781800	-1.93178600
S	-2.04676000	5.82356200	-1.47098800
H	-2.49840100	7.07830800	-1.31470800
N	-0.32984300	-0.05640200	-1.74457200
C	-0.17435600	0.99878500	-2.51742900
C	-0.29823000	-1.16020600	-2.57277600
H	-0.14976400	2.02915800	-2.18875500
C	-0.12171800	-0.75782000	-3.85788600
H	-0.40469600	-2.16438400	-2.18696300
H	0.08630900	1.24577100	-4.58021100
C	6.37193900	-3.21170700	-0.37644900
O	6.94174600	-3.44663200	0.66461600
O	7.01530500	-3.15234900	-1.54985800
C	6.72970200	2.19071900	-0.43043700
O	7.33230700	2.33956300	0.60803800
O	7.35213500	2.05834200	-1.60913800
N	-0.04596800	0.61769100	-3.79440300

C	-0.82024200	1.05256800	2.93141500
C	-1.12007500	-0.67528600	4.27687500
C	-0.99111900	-1.10310600	2.99426600
N	-0.80470200	-0.01705500	2.16298100
H	-0.70019400	2.07520700	2.59963400
H	-1.05750900	1.34095900	4.99146100
H	-1.01916000	-2.11395300	2.61209700
N	-1.00754700	0.69743700	4.20860500
C	-0.01748200	-1.51868400	-5.13363100
H	-0.81455600	-1.23724200	-5.83186300
H	0.94424800	-1.33772100	-5.62801600
H	-0.10190400	-2.59077000	-4.93437900
C	-1.33668700	-1.40946100	5.55408400
H	-0.51543200	-1.23413400	6.25890800
H	-2.26998100	-1.09960600	6.03874900
H	-1.39466700	-2.48404700	5.35898900
H	7.96103000	-3.32748000	-1.37700900
H	8.31368700	2.10720300	-1.44193200

Heme c (Red, Protonated)

Fe	-0.55949700	-0.02992700	0.21387700
C	-4.16199100	-2.31321100	-0.21083000
C	-3.27031800	-3.33580400	-0.09812800
C	-1.96937200	-2.71648900	0.05947900
C	-3.38606700	-1.08500300	-0.13241400
N	-2.06506200	-1.36457700	0.03514000
C	-3.91227700	0.19548300	-0.21565400
C	-3.20737600	1.38450000	-0.14110400
N	-1.86126200	1.50016500	0.03003000
C	-1.59663800	2.82892500	0.05547600
C	-3.82811700	2.69216800	-0.23174800
C	-2.82297400	3.59968900	-0.10301300
C	-0.32018300	3.37073800	0.20417100
C	0.84748400	2.65190200	0.35750300
N	0.94195800	1.29309500	0.39044500
C	3.04170600	2.24206800	0.63715800
C	2.16241300	3.26352200	0.50877000
C	2.08801400	-1.44924600	0.56947100
N	0.74841900	-1.54883600	0.39959100
C	0.47046200	-2.88378500	0.37747400
C	1.68887500	-3.66646400	0.53911400
C	2.69879500	-2.77227800	0.65982200
C	-0.78473200	-3.43190700	0.22010600
C	2.79816000	-0.25971000	0.64905900

C	2.25724600	1.01286600	0.55957500
H	-4.98607500	0.28694100	-0.35356800
H	-0.22985800	4.45212000	0.18593600
H	-0.85608000	-4.51697900	0.22243100
H	3.87281200	-0.33227600	0.79575500
C	4.53148400	2.30915000	0.79430700
H	4.87833100	1.53304100	1.48636100
H	4.81419300	3.26459800	1.24950300
C	5.25931300	2.15766300	-0.53979900
H	4.96346800	2.94883900	-1.24014600
H	4.99926000	1.21091100	-1.03043700
C	4.16586100	-3.03922600	0.81893300
H	4.61442700	-2.31240100	1.50610600
H	4.31699800	-4.02100000	1.28078200
C	4.90709200	-2.99614100	-0.51578300
H	4.77423900	-2.02744300	-1.01427200
H	4.50879400	-3.74691600	-1.20990300
C	1.74869600	-5.16122700	0.55133400
H	1.11214500	-5.58306600	1.33865100
H	1.40834500	-5.58337200	-0.40230400
H	2.76894000	-5.51702100	0.72278300
C	2.42354200	4.73648900	0.50780900
H	2.12977500	5.19458700	-0.44475700
H	1.86061100	5.24543300	1.29982500
H	3.48471700	4.95241500	0.66316000
C	-2.97414300	5.09572700	-0.08653400
H	-4.02726500	5.31387700	-0.27894200
C	-5.29608400	2.91682200	-0.42005200
H	-5.65842700	2.43395000	-1.33554500
H	-5.87402400	2.50403400	0.41588400
H	-5.54123400	3.97981600	-0.49298700
C	-5.65718400	-2.37591400	-0.36578200
H	-6.04630400	-1.35600700	-0.39717300
C	-3.50213700	-4.81629000	-0.11747300
H	-2.77400400	-5.32315800	-0.76095800
H	-3.40695700	-5.25025200	0.88636600
H	-4.49676300	-5.06196800	-0.49621200
C	-6.36148200	-3.10464300	0.77675400
H	-6.11799000	-2.61562400	1.72760200
H	-7.44903500	-3.07134300	0.64661800
H	-6.05662900	-4.15286000	0.84279400
C	-2.60247600	5.74605700	1.24569200
H	-2.81210700	6.82131000	1.22181300
H	-3.20145500	5.30089500	2.04919500

H	-1.54573100	5.60999500	1.49239500
S	-6.04566600	-3.10390000	-2.00721900
H	-7.37725700	-2.93613200	-1.95975900
S	-2.05815200	5.82965500	-1.50207300
H	-2.52644100	7.08084900	-1.36818700
N	-0.29782500	-0.05360500	-1.80319700
C	-0.11179400	0.98930600	-2.57555800
C	-0.27733700	-1.15320800	-2.63483900
H	-0.07143400	2.02023100	-2.24671900
C	-0.07617500	-0.76141000	-3.92469100
H	-0.40898800	-2.15571200	-2.24835100
H	0.18123800	1.23371200	-4.64917200
C	6.38356500	-3.23539400	-0.38740700
O	6.98684800	-3.43215200	0.64718500
O	7.00643700	-3.20953900	-1.57482400
C	6.75454900	2.19997700	-0.41188500
O	7.37871500	2.30901600	0.62327900
O	7.36830400	2.10016200	-1.60006500
N	0.02700300	0.60981300	-3.86541500
C	-0.83248400	1.05330900	3.00161200
C	-1.16017200	-0.66588100	4.35205500
C	-1.02747600	-1.08677700	3.06244700
N	-0.82356200	-0.00657500	2.23008500
H	-0.69999000	2.07620200	2.67192400
H	-1.07990100	1.34497300	5.07479900
H	-1.06440200	-2.09751000	2.67670300
N	-1.03268800	0.70322500	4.29170700
C	0.02820800	-1.52995300	-5.19730800
H	-0.75304100	-1.23693100	-5.90848500
H	0.99956800	-1.37472900	-5.68144300
H	-0.08227400	-2.59888800	-4.99420800
C	-1.39159100	-1.40493500	5.62536800
H	-0.57399500	-1.24326800	6.33764000
H	-2.32509500	-1.09188500	6.10755000
H	-1.45786700	-2.47775300	5.42356300
H	8.33219400	2.13036800	-1.45710800
H	7.95799200	-3.36623600	-1.43186000

Heme c (Ox. Deprotonated)

Fe	-0.52429900	-0.02175800	0.20621900
C	-4.12389200	-2.31629200	-0.19227400
C	-3.24414200	-3.33321500	-0.07419000
C	-1.92449700	-2.71271200	0.07982600
C	-3.33235600	-1.07989500	-0.12123500

N	-2.02908600	-1.34507700	0.04371100
C	-3.87487000	0.20912200	-0.21168300
C	-3.17532500	1.38359300	-0.14273900
N	-1.81882700	1.50580700	0.02396500
C	-1.56735300	2.82014400	0.04327200
C	-3.79813200	2.70384400	-0.23337300
C	-2.79654700	3.60288200	-0.10997000
C	-0.27364300	3.37718800	0.18213100
C	0.88066900	2.67493400	0.32827000
N	0.98064000	1.29113400	0.36657900
C	3.07866400	2.26098300	0.58952300
C	2.20731500	3.28065900	0.46815700
C	2.14379800	-1.43017600	0.54885000
N	0.76932200	-1.53963600	0.38916100
C	0.51323800	-2.84303500	0.38550700
C	1.73648900	-3.64329600	0.54572800
C	2.75210000	-2.76773900	0.64663700
C	-0.76633200	-3.41702700	0.23928200
C	2.83396900	-0.26812000	0.61265800
C	2.27192600	1.03293800	0.51909200
H	-4.94822000	0.29385200	-0.34708700
H	-0.19426700	4.45862600	0.15252500
H	-0.82675400	-4.50134200	0.25274300
H	3.91019000	-0.32722400	0.74506200
C	4.56840700	2.30143800	0.72425000
H	4.90795400	1.55581100	1.45339700
H	4.87879100	3.26806300	1.13418500
C	5.29663700	2.07606300	-0.59993000
H	4.96958700	2.82323700	-1.33684200
H	5.02734900	1.10189400	-1.03260100
C	4.21881100	-3.03121900	0.77390200
H	4.67782800	-2.31193400	1.46274600
H	4.38366300	-4.01500700	1.22542000
C	4.94852700	-2.97807400	-0.56796100
H	4.81769100	-1.99454400	-1.04014800
H	4.49975700	-3.70004700	-1.26502100
C	1.76537400	-5.13623400	0.57072100
H	1.12765200	-5.53685100	1.36899400
H	1.40591100	-5.55882000	-0.37635100
H	2.78132000	-5.50707800	0.73561000
C	2.46396600	4.75139100	0.46225700
H	2.16340000	5.20383400	-0.49128300
H	1.90126100	5.25894000	1.25581200
H	3.52576000	4.96665600	0.61321200

C	-2.93321400	5.09959500	-0.08402100
H	-3.98556100	5.32905200	-0.26833600
C	-5.26455700	2.91852000	-0.41552700
H	-5.62229100	2.43116500	-1.33069500
H	-5.83190500	2.49669100	0.42331600
H	-5.51791900	3.97972100	-0.48573700
C	-5.61985700	-2.37145100	-0.34667300
H	-6.00544400	-1.35170400	-0.40875000
C	-3.46206600	-4.81169500	-0.08143600
H	-2.76783700	-5.30590100	-0.77128600
H	-3.29177700	-5.23882600	0.91526500
H	-4.47627800	-5.06884900	-0.39415400
C	-6.31951600	-3.06115300	0.82259600
H	-6.07190200	-2.54282400	1.75690100
H	-7.40740500	-3.02885300	0.69439600
H	-6.01727200	-4.10801300	0.91975100
C	-2.54956500	5.72473600	1.25684000
H	-2.74451100	6.80300600	1.24700900
H	-3.15438200	5.27749400	2.05488900
H	-1.49400800	5.57317900	1.50198700
S	-6.00504600	-3.15116000	-1.96224300
H	-7.33543300	-2.97242500	-1.92101400
S	-2.00819800	5.82701000	-1.49383500
H	-2.39239100	7.09831000	-1.29714300
N	-0.27625400	-0.05880500	-1.81249400
C	-0.06564800	0.97226000	-2.59682900
C	-0.28850000	-1.16412500	-2.63581200
H	0.00498400	2.00523800	-2.28178700
C	-0.08246300	-0.78879300	-3.92812500
H	-0.44562700	-2.16024100	-2.24366900
H	0.22493100	1.19147500	-4.66974100
C	6.46168900	-3.27304000	-0.48682000
O	6.93063300	-3.57343900	0.63670500
O	7.08437200	-3.18767100	-1.57256800
C	6.83590900	2.14457300	-0.50024600
O	7.32834300	2.39144000	0.62632600
O	7.45442200	1.94797900	-1.57376600
N	0.05675500	0.57877000	-3.87996200
C	-0.74504800	1.06666000	3.00300000
C	-1.10979400	-0.64615100	4.34736300
C	-1.00033600	-1.06573300	3.05680200
N	-0.77273600	0.00977800	2.22529900
H	-0.58577000	2.08755100	2.68109600
H	-0.96561200	1.35816500	5.07517700

H	-1.07078300	-2.07421900	2.67120000
N	-0.94400800	0.71804100	4.28951500
C	-0.00256500	-1.57139300	-5.19338700
H	-0.77662300	-1.26234800	-5.90621800
H	0.97179500	-1.44695300	-5.68122600
H	-0.14211600	-2.63563700	-4.98173600
C	-1.35109500	-1.38403200	5.61900800
H	-0.52312800	-1.24805600	6.32521400
H	-2.27099200	-1.04602700	6.11125600
H	-1.44978000	-2.45422800	5.41458700

Heme c (Red, Deprotonated)

Fe	-0.52395000	-0.03011900	0.20913100
C	-4.13633400	-2.30145500	-0.19954500
C	-3.24615000	-3.32709700	-0.09170500
C	-1.94374800	-2.71168300	0.05988200
C	-3.35760000	-1.07624900	-0.12398500
N	-2.03558000	-1.36044300	0.03690700
C	-3.87953300	0.20634600	-0.20268300
C	-3.17129600	1.39348300	-0.12958500
N	-1.82278300	1.50495300	0.03500700
C	-1.55395700	2.83173700	0.06240800
C	-3.78740000	2.70243800	-0.21355300
C	-2.77777600	3.60670700	-0.08743200
C	-0.27288700	3.36940600	0.20560800
C	0.89235200	2.64747300	0.35021300
N	0.98282900	1.28730100	0.37906700
C	3.09061100	2.23095900	0.61403000
C	2.21151500	3.25348800	0.49489700
C	2.12144100	-1.45820600	0.54598400
N	0.78048700	-1.55244000	0.38547700
C	0.49808900	-2.88761100	0.36548300
C	1.71651300	-3.67298500	0.52155500
C	2.73159000	-2.78405300	0.63558100
C	-0.75829200	-3.43114100	0.21470500
C	2.83647200	-0.27087400	0.61915400
C	2.29877200	1.00375200	0.53717000
H	-4.95386700	0.30136500	-0.33506700
H	-0.17921900	4.45057800	0.18913300
H	-0.83286900	-4.51607100	0.21713200
H	3.91265700	-0.34754700	0.74988000
C	4.58232500	2.29446700	0.75762200
H	4.93437000	1.50133000	1.42713800
H	4.86628500	3.23590300	1.24089600

C	5.30912200	2.18369300	-0.58221700
H	4.97571400	2.98415400	-1.25585800
H	5.04137800	1.24462000	-1.08477400
C	4.19901600	-3.05716300	0.78479700
H	4.65736800	-2.31583700	1.44970500
H	4.34623800	-4.02514600	1.27604600
C	4.93733700	-3.06020200	-0.55335700
H	4.80905400	-2.09554400	-1.06170300
H	4.49226900	-3.80810100	-1.22339000
C	1.77279200	-5.16814800	0.53482900
H	1.13683000	-5.58972800	1.32306200
H	1.43118900	-5.59156600	-0.41805300
H	2.79355700	-5.52311100	0.70521000
C	2.47811500	4.72576300	0.49632100
H	2.18499600	5.18814800	-0.45462300
H	1.91973900	5.23687400	1.29046100
H	3.54114200	4.93415900	0.64964600
C	-2.92451800	5.10313000	-0.06604600
H	-3.97844000	5.32499600	-0.24982300
C	-5.25560100	2.93367600	-0.39367100
H	-5.62528200	2.45339900	-1.30762600
H	-5.83113300	2.52274100	0.44488900
H	-5.49660200	3.99782400	-0.46435500
C	-5.63236600	-2.36053800	-0.34712700
H	-6.01917900	-1.33963000	-0.37547400
C	-3.48275500	-4.80706100	-0.11102700
H	-2.75792600	-5.31613700	-0.75655400
H	-3.38654800	-5.24235000	0.89221000
H	-4.47913100	-5.04952100	-0.48742900
C	-6.33316800	-3.08916100	0.79760300
H	-6.08392900	-2.60208800	1.74797800
H	-7.42130000	-3.05321300	0.67283800
H	-6.03034700	-4.13815500	0.86077600
C	-2.54066900	5.74989700	1.26443600
H	-2.74705700	6.82589500	1.24442200
H	-3.13484600	5.30496500	2.07165900
H	-1.48253600	5.60985200	1.50274300
S	-6.03136500	-3.08555700	-1.98758700
H	-7.36241100	-2.91536200	-1.93309300
S	-2.01765400	5.83839800	-1.48698900
H	-2.48042500	7.09090300	-1.34596600
N	-0.27290300	-0.05151400	-1.80929000
C	-0.08166400	0.99160600	-2.57985800
C	-0.26182600	-1.14957000	-2.64298600

H	-0.03286500	2.02137000	-2.24842600
C	-0.06093100	-0.75666300	-3.93259300
H	-0.39859800	-2.15179800	-2.25744800
H	0.20913000	1.23798300	-4.65378400
C	6.44214900	-3.34735800	-0.48652400
O	6.96438200	-3.58605900	0.63354100
O	7.05564800	-3.32613200	-1.59072800
C	6.83977200	2.24527000	-0.51865300
O	7.39640700	2.34552900	0.60571400
O	7.43912400	2.18770700	-1.62937500
N	0.05142700	0.61378600	-3.87099800
C	-0.77311400	1.04997800	3.00025600
C	-1.10263500	-0.66905600	4.35057100
C	-0.98158400	-1.08879800	3.05938700
N	-0.77640100	-0.00872600	2.22729900
H	-0.63569600	2.07228500	2.67066400
H	-1.00377900	1.34016100	5.07578300
H	-1.02707600	-2.09856800	2.67194900
N	-0.96681700	0.69936500	4.29141200
C	0.03629600	-1.52341200	-5.20689000
H	-0.74405500	-1.22388600	-5.91637700
H	1.00795500	-1.37379100	-5.69221700
H	-0.08090200	-2.59199600	-5.00565800
C	-1.32948300	-1.40856800	5.62448600
H	-0.50568600	-1.25326600	6.33103400
H	-2.25744000	-1.09044900	6.11405500
H	-1.40398200	-2.48065200	5.42157300

Supporting Information 8: The Detailed data for Section 3.3.3.

* Sample input for C-PCM calculation: (B3LYP, Assumed eps=10.0) *

#p opt ub3lyp/genecp scrf=(cpcm,read) int=grid=ultrafine

Protonted, Oxidized Eps=10.0

1 2

Fe	-0.56177600	-0.03245800	0.21842600
C	-4.18772800	-2.33443900	-0.20064800
C	-3.28648900	-3.36503300	-0.08553800
C	-1.98021000	-2.74371400	0.07052600
C	-3.41034500	-1.09855100	-0.12568600
N	-2.08022600	-1.37772200	0.04284300
C	-3.93985700	0.18908000	-0.20877000
C	-3.23419300	1.38896900	-0.13333100
N	-1.87986700	1.50612200	0.03899700
C	-1.61234600	2.84944600	0.06825200
C	-3.85814600	2.70044500	-0.22224700
C	-2.84646400	3.61891700	-0.08834900
C	-0.33088700	3.38917300	0.21456000
C	0.85477200	2.67104700	0.36235400
N	0.95155600	1.30373500	0.39063900
C	3.06218200	2.25497300	0.63295600
C	2.16875700	3.28539200	0.51099000
C	2.11492700	-1.46119400	0.56478500
N	0.75995100	-1.56252600	0.39884300
C	0.48199800	-2.90550500	0.38152900
C	1.70140700	-3.68845500	0.54147700
C	2.72412800	-2.78555900	0.65658100
C	-0.78867000	-3.45449000	0.22958000
C	2.82034800	-0.26021400	0.64156900
C	2.28188300	1.02327300	0.55466900
H	-5.01191100	0.27791300	-0.34620000
H	-0.24417500	4.46879300	0.19751500
H	-0.85823400	-4.53740200	0.23589900
H	3.89351500	-0.33137200	0.78706100
C	4.55666600	2.33228600	0.77966100
H	4.91614500	1.56490200	1.47500500
H	4.84088200	3.29323800	1.22150300
C	5.29454900	2.17046600	-0.56076000
H	4.98396600	2.94527800	-1.27262600
H	5.04254600	1.21405800	-1.03595200

C	4.19480200	-3.05998500	0.80742100
H	4.65194800	-2.34190000	1.49810000
H	4.34822100	-4.04692600	1.25670500
C	4.94917900	-3.00735300	-0.53260900
H	4.82520100	-2.03045700	-1.01642400
H	4.54034000	-3.74102400	-1.23853500
C	1.76466000	-5.18739600	0.56132400
H	1.09245400	-5.61193300	1.31780800
H	1.46958500	-5.61987200	-0.40392100
H	2.77589100	-5.54191000	0.78240600
C	2.43184800	4.76249000	0.51827400
H	2.18097800	5.22527100	-0.44534700
H	1.83489400	5.27730100	1.28191600
H	3.48482800	4.97986800	0.72089400
C	-3.00786400	5.11448200	-0.06290400
H	-4.05842600	5.34015200	-0.25532600
C	-5.32966500	2.92674000	-0.41161700
H	-5.69563000	2.44150500	-1.32521500
H	-5.91204500	2.51491400	0.42271600
H	-5.57534900	3.98965800	-0.48639800
C	-5.68397600	-2.39696300	-0.35713900
H	-6.08224300	-1.38161800	-0.38082500
C	-3.52078500	-4.84929000	-0.10175500
H	-2.76762700	-5.36417300	-0.70950000
H	-3.46529500	-5.28044200	0.90723300
H	-4.50008300	-5.09772000	-0.51665200
C	-6.40805800	-3.16122700	0.75920500
H	-6.17792000	-2.69500000	1.72503000
H	-7.49317000	-3.12389700	0.61481400
H	-6.10355100	-4.20965200	0.80377700
C	-2.61741100	5.77862800	1.26587600
H	-2.82070400	6.85435700	1.23561300
H	-3.21442900	5.34245800	2.07613800
H	-1.56099000	5.63477500	1.50546900
S	-6.07094300	-3.10325500	-2.05427800
H	-7.41310900	-2.95528800	-1.99950000
S	-2.08712600	5.87134600	-1.51709800
H	-2.56054300	7.12751900	-1.36662000
N	-0.30505200	-0.05251900	-1.83195200
C	-0.11615100	1.00162800	-2.60905100
C	-0.29271600	-1.15159400	-2.67315300
H	-0.07024100	2.02755300	-2.27898200
C	-0.09410600	-0.75614800	-3.97310000
H	-0.42689400	-2.15053700	-2.28774900

H	0.16849900	1.24545100	-4.68707600
C	6.43147400	-3.27209900	-0.38976300
O	7.01873400	-3.50263000	0.64922200
O	7.06175600	-3.22417100	-1.58494300
C	6.79908500	2.23907500	-0.42123200
O	7.41387300	2.38237000	0.61752500
O	7.41494200	2.11956100	-1.61885500
N	0.01536300	0.62190300	-3.90597000
C	-0.83349900	1.05948700	3.04664500
C	-1.15190400	-0.67178000	4.40760700
C	-1.01956300	-1.09304700	3.10728700
N	-0.82186900	-0.01028700	2.26799600
H	-0.70715200	2.07918600	2.71828200
H	-1.07755700	1.34491500	5.12419000
H	-1.05233500	-2.09988500	2.72053600
N	-1.02961800	0.70526100	4.34258200
C	0.00144200	-1.52490000	-5.25014700
H	-0.77994800	-1.23074900	-5.96180400
H	0.96972800	-1.37523800	-5.74369500
H	-0.11202800	-2.59356100	-5.04874000
C	-1.37704300	-1.41542500	5.68323700
H	-0.56055600	-1.25415500	6.39796100
H	-2.30947700	-1.10972600	6.17374000
H	-1.43966100	-2.48801800	5.48053300
H	8.37666900	2.17145800	-1.45461600
H	8.00799100	-3.40222200	-1.41905100

Fe 0
SDD
****
S,O,N,C,H 0
6-31++G(d,p)
****

Fe 0
SDD

Eps=10.0

Note: This simulation is not assumed to the real compounds. (The parameter used in PCM calculation except for ε (dielectric constant) is set to "water solvent". We just changed the parameter ε in this case.)
